# Emergence of Oropouche Virus in Espírito Santo State, Brazil, 2024

**DOI:** 10.3201/eid3106.241946

**Published:** 2025-06

**Authors:** Edson Delatorre, Gabriela Colombo de Mendonça, Felipe Donateli Gatti, Anna Clara Gregório Có, Julia del Piero Pereira, Eric Arrivabene Tavares, Joana Zorzal Nodari, Agata Rossi, Suwellen Sardinha Dias de Azevedo, Cláudio Tavares Sacchi, Karoline Rodrigues Campos, Adriana Bugno, Lyvia Neves Rebello Alves, Lucas André Silva Bonela, Jaqueline Pegoretti Goulart, Thiago de Jesus Sousa, Felipe Gomes Naveca, Rodrigo Ribeiro-Rodrigues

**Affiliations:** Universidade Federal do Espírito Santo Centro de Ciências da Saúde, Vitória, Brazil (E. Delatorre, A. Rossi, R. Ribeiro-Rodrigues); Laboratório Central de Saúde Pública do Estado do Espírito Santo, Vitória (G.C. de Mendonça, F.D. Gatti, A.C.G. Có, J.P. Pereira, E.A. Tavares, J.Z. Nodari, S.S.D. de Azevedo, L.N.R. Alves, L.A.S. Bonela, J.P. Goulart, T.J. Sousa); Instituto Adolfo Lutz, São Paulo, Brazil (C.T. Sacchi, K.R. Campos, A. Bugno); Instituto Oswaldo Cruz, Fiocruz, Rio de Janeiro, Brazil (F.G. Naveca); Instituto Leônidas e Maria Deane, Fiocruz, Manaus, Brazil (F.G. Naveca)

**Keywords:** Oropouche virus, viruses, disease outbreaks, climate, arboviruses, symptoms, epidemiology, vector-borne infections, Brazil

## Abstract

Oropouche virus (OROV), historically endemic to the Amazon, had spread to nearly all Brazil states by 2024; Espírito Santo emerged as a transmission hotspot in the Atlantic Forest biome. We characterized the epidemiologic factors driving OROV spread in nonendemic southeast Brazil, analyzing environmental and agricultural conditions contributing to viral transmission. We tested samples from 29,080 suspected arbovirus-infected patients quantitative reverse transcription PCR for OROV and dengue, chikungunya, Zika, and Mayaro viruses. During March‒June 2024, the state had 339 confirmed OROV cases, demonstrating successful local transmission. Spatial analysis revealed that most cases clustered in municipalities with tropical climates and intensive cacao, robusta coffee, coconut, and pepper cultivation. Phylogenetic analysis identified the Espírito Santo OROV strains as part of the 2022–2024 Amazon lineage. The rapid spread of OROV outside the Amazon highlights its adaptive potential and public health threat, emphasizing the need for enhanced surveillance and targeted control measures.

Oropouche virus (OROV), classified as *Orthobunyavirus oropoucheense*, family *Peribunyaviridae*, is an arthropodborne virus with a negative-sense RNA genome consisting of 3 segments, large (L), medium (M), and small (S) (*1*). A neglected arbovirus, OROV causes Oropouche fever and circulates primarily in Central America, South America, and the Caribbean (*2*–*4*). In Brazil, OROV was historically confined to the Amazon basin, where several vector species and a range of reservoirs maintain its sylvatic transmission cycle (*2*,*3*,*5*). In urban areas in the Amazon, *Culicoides paraensis*, a midge commonly found in tropical, humid environments rich in organic matter, such as forests and plantations, is the primary vector responsible for OROV transmission to humans (*3*,*4*). Humans might acquire OROV infection in forested regions and subsequently introduce it to urban settings. The widespread distribution of the vector, coupled with increased human mobility and the influence of climate change, might enable the virus’s gradual expansion beyond its historical range in Brazil, raising concerns about the potential for broader geographic spread (*6*,*7*).

Since the 1960s, occasional OROV spillovers to humans have led to >30 documented localized outbreaks or large-scale epidemics in the Amazon basin, underscoring the virus’s epidemic potential (*8*–*12*). Although incidence is highest in the Amazon, sporadic cases have been reported in other states in Brazil without leading to widespread outbreaks (*13*). 

During August 2022‒March 2024, a new outbreak triggered by a reassortant OROV lineage emerged in Brazil’s western Amazon region, causing ≈6,000 reported cases (*14*). In 2024, that reassortant lineage led to the largest recorded outbreak outside the virus’s endemic zone; OROV was detected in all regions of Brazil (*15*,*16*). Outside the Amazon, high incidence rates were observed in the Atlantic Forest region, particularly in municipalities with low population densities and agricultural activities favoring the establishment of *C. paraensis* vector populations, such as cocoa and banana cultivation (*17*).

OROV infections typically manifest as an acute febrile illness characterized by headache, myalgia, and arthralgia (*14*). Those symptoms overlap with those of infection with other endemic arboviruses, such as dengue virus (DENV), Zika virus (ZIKV), and chikungunya virus (CHIKV) (*2*,*3*). However, emerging evidence has linked OROV to fatal cases (*18*). Unprecedented vertical transmission was also reported, and some cases resulted in congenital anomalies or fetal death (*19*,*20*). Therefore, the significant shift in OROV’s pathogenicity marks a new epidemiologic paradigm for Oropouche fever.

Espírito Santo state, located in southeastern Brazil and entirely within the Atlantic Forest biome, has emerged as a major hotspot for OROV transmission outside the Amazon region, recording the highest state-level incidence rate among non-Amazon states in 2024 (*21*). The state’s extensive agricultural activities, particularly in coffee, cocoa, and banana cultivation (*22*), combined with high rural worker mobility and environmental conditions favorable for *C. paraensis* midge establishment, may have contributed to viral spread. The first case of Oropouche infection in Espírito Santo was detected on March 24, 2024. We used epidemiologic and genomic approaches to analyze the emergence and dissemination of the new OROV variant in Espírito Santo state. We also examined the regional characteristics that enable its transmission and contribute to its establishment in this previously unaffected area.

## Materials and Methods

### Study Population

Our study analyzed samples from patients who visited public health units in Espírito Santo state with arboviral-like symptoms (ZDC: Zika, dengue, and chikungunya) during February 25–June 15, 2024. All suspected cases of acute arboviral infections in Espírito Santo are centralized at the state’s central laboratory, Laboratório Central de Saúde Pública do Espírito Santo (LACEN-ES, Vitória, Brazil), which receives samples from all 78 municipalities for diagnostic testing. During the study period, a total of 29,080 samples were tested, corresponding to ≈0.76% of the state’s population (3,833,712 inhabitants). The serum or plasma samples were subsequently sent to the LACEN-ES for viral molecular diagnosis. The Human Research Ethics Committee at the University of Vila Velha (Vila Velha, Brazil) approved this study (Certificate of Presentation for Ethical Assessment no. 84698324.7.0000.5064) and waived the need for written informed consent. The committee is registered with Brazil’s National Research Ethics Commission and oversees studies involving public health institutions in Espírito Santo, including the state’s Central Laboratory where this investigation was conducted.

### Sample Processing and Quantitative Reverse Transcription PCR

We processed samples for nucleic acid extraction using magnetic bead-based systems TANBead Maelstrom 9600 (Taiwan Advanced Nanotech Inc., https://www.tanbead.com), EXTRACTA 96 (Loccus, https://www.loccus.com.br) and TechStar YC-702 (Wuxi Techstar Technology Co., http://www.techstarbio.com), following manufacturers’ instructions. Subsequently, for molecular testing, we used 3 different quantitative reverse transcription (qRT-PCR) kits: Molecular ZCD Tipagem Bio-Manguinhos (https://www.bio.fiocruz.br), Biomol ZDC (IBMP, https://www.ibmp.org.br), and VIASURE Zika, Dengue & Chikungunya (ThermoFisher, https://www.thermofisher.com), following the manufacturers’ protocols. For the samples not detectable for ZDC, we performed a multiplex qRT-PCR to investigate for OROV and Mayaro virus (MAYV), as described by Naveca et al. (*23*). To extend testing, we adapted the laboratory diagnosis to carry out the procedure on a pool of 8 samples. After detecting the target, we conducted another q RT-PCR with the 8 samples individually to confirm the test.

### Epidemiologic Data and Environmental Context

We retrieved individual-level of OROV-positive case data from the eSUS Brazil Ministry of Health (https://sisaps.saude.gov.br/sistemas/esusaps) and Gerenciador de Ambiente Laboratorial (GAL; http://gal.datasus.gov.br) systems at the municipal level for the state of Espírito Santo, covering cases reported through June 15, 2024. We linked each case to anonymized metadata, including demographics, location, symptoms (fever, headache, myalgia, retroorbital pain, back pain, arthritis, petechiae, rash, arthralgia, nausea, conjunctivitis, vomiting, and leukopenia), and hospitalization and notification/symptom dates.

We calculated the municipal incidence of OROV on the basis of the 2022 census data from the Brazilian Institute of Geography and Statistics (IBGE) (*24*). Estimated incidence mapping used the geoBR package in RStudio (http://www.rstudio.com) with IBGE municipal boundary shapefiles (*25*). We obtained data about the agricultural establishments at the municipal level from the 2017 IBGE census of agriculture (*26*). We estimated the Spearman correlation for all municipalities reporting cases to explore the relationship between the planted area of the top 10 crops in Espírito Santo and the number of OROV cases. In addition, we analyzed between-group differences in cycle threshold (Ct) values (viral load proxy) for each symptom using 2-tailed Mann-Whitney U tests (α = 0.05).

### Generation Time and Instantaneous Reproduction Number Estimation

The generation time represents the interval between successive rounds of infection. Although that interval has been estimated for other arboviruses, no such estimates exist for OROV. We estimated the generation time using a combination of human viral clearance data (*27*), mosquito mortality rates (*28*), and data from experimental studies involving vector competence (*29*), using a framework applied previously for ZIKV (*30*) and MAYV (*31*). We conducted parameter inference using a Bayesian framework implemented with Markov chain Monte Carlo methods (Appendix Figures 1, 2). We then used the posterior distributions of the generation time parameters (Appendix Tables 1, 2) to inform the calculation of the reproduction number. We estimated the instantaneous reproduction number (R_t_) for OROV using the R package EpiEstim (*32*). We reconstructed the R_t_ for the April–June period using a Bayesian inference model with a sliding time window of τ = 7 days (Appendix).

### Sample Selection and Next-Generation Sequencing

We selected 7 samples with Ct <27 for whole-genome sequencing from a pool of all OROV-positive samples identified through RT-PCR during the study. To ensure geographic representativeness, we chose samples from the municipalities of Colatina, Rio Bananal, and Laranja da Terra, which were among the most affected regions in the state. We sequenced 6 samples at Instituto Adolfo Lutz (IAL), yielding complete S and M segments (Appendix Table 3). We sequenced 1 sample at LACEN/ES using Illumina RNA Prep with Enrichment Tagmentation protocol (https://www.illumina.com) with the Respiratory Pathogen ID/AMR Enrichment Panel Kit (Illumina); we extracted OROV reads from the nontarget portion of the kit. We conducted genome assembly using a custom version of the ViralFlow pipeline version 1.0.1 (*33*), referencing GenBank sequences NC_005776.1 (L segment), NC_005775.1 (M segment), and NC_005777.1 (S segment).

### Bioinformatics and Phylogenetic Inference

For the phylogenetic analysis, we aligned 7 new sequences from Espírito Santo with 145 OROV strains sampled in the Americas (1955–2023) and available in GenBank as of August 2024. The alignment, performed in MAFFT version 7 (https://mafft.cbrc.jp) (*34*), included the prototypical viruses Iquitos, Madre de Dios, and Perdões as outgroups. We selected the generalized time-reversible discrete gamma substitution model in jModelTest2 (https://github.com/ddarriba/jmodeltest2). We used phylogenetic inference in MrBayes version 3.2.7a (*35*) to sample trees until parameter convergence (effective sample size >200), with node support determined by posterior probabilities from the majority-rule consensus topology.

## Results

**Figure 1:**
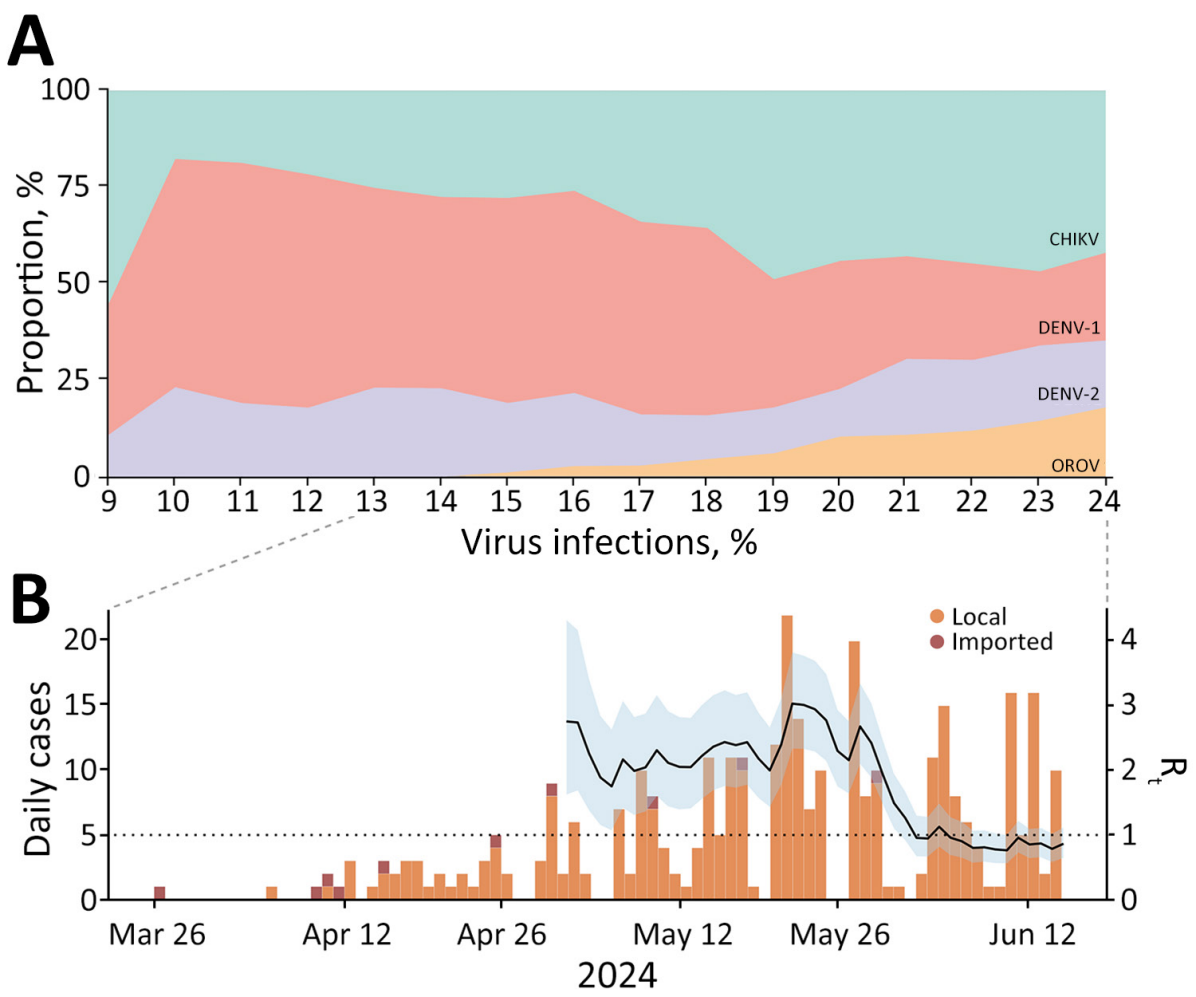
Temporal distribution and reproduction number of Oropouche virus in Espírito Santo, Brazil. A) Weekly percentages of CHIKV, DENV-1, DENV-2, and OROV infections for epidemiologic weeks 9–24, 2024. B) Estimates of R_t_ during the OROV outbreak. Solid black curve represents the median R_t_; shaded area indicates 95% credible interval, based on daily incidence data including local and imported cases. Dotted line indicates R_t_ = 1. R_t_ was estimated using sliding windows of τ = 7 days. CHIKV, chikungunya virus; DENV, dengue virus; OROV, Oropouche virus; R_t_, instantaneous reproduction number.

Approximately 29,100 patients in Espírito Santo experiencing arbovirus-like illness were tested by real-time RT-PCR for the presence of DENV-1, DENV-2, CHIKV, ZIKV, OROV, and MAYV during March–June 2024. Until epidemiologic week 13, DENV-1 represented ≈50% of the positive cases, followed by CHIKV and DENV-2 (Figure 1). However, from epidemiologic week 13 onward, OROV cases were detected, marking a pronounced shift in the epidemiologic landscape. OROV cases increased rapidly, reaching 339 cases within 10 weeks. By epidemiologic week 24, the frequency of OROV infections approached the levels of DENV-1, DENV-2, and CHIKV, indicating a comparable circulation of these viruses at the outbreak’s peak. After the emergence of OROV, the proportion of CHIKV cases also rose, eventually surpassing that of DENV. Initially, OROV cases were primarily classified as imported; however, community spread became evident as local transmission was established. During epidemiologic weeks 18–27, the time-varying R_t_ for OROV remained ≈2.5 (95% credible interval ≈1.5–3.0), we observed a peak value of ≈3.0 in epidemiologic week 26. After this peak, a marked reduction in R_t_ occurred, and it was below 1.0 by epidemiologic week 28, indicating a decline in transmission and a shift toward containment of the outbreak (Figure 1).

**Figure 2:**
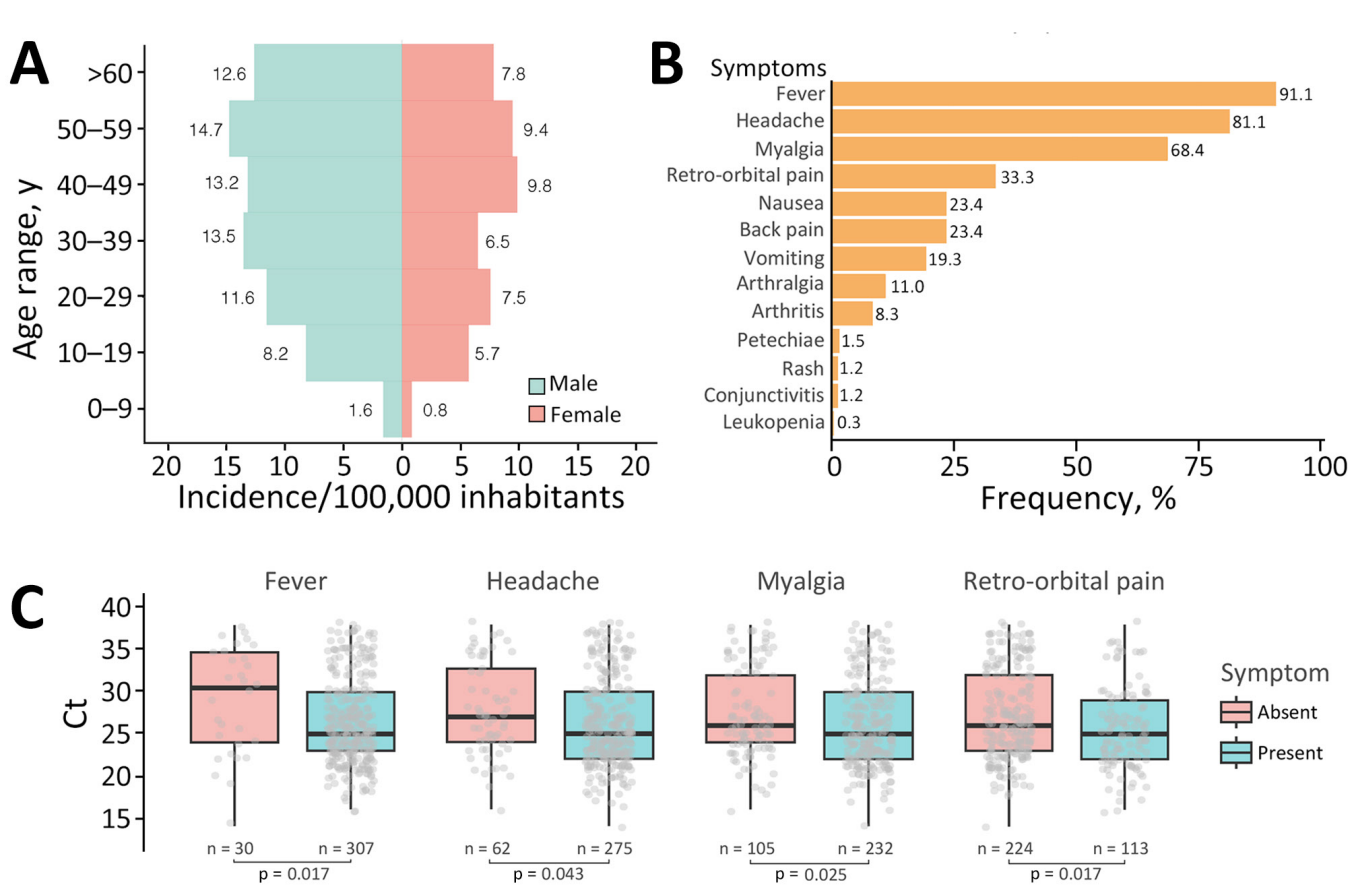
Demographics, symptoms, and Ct value comparison in OROV-positive patients from Espírito Santo, Brazil. A) Age-sex pyramid of the OROV incidence rate (per 100,000 inhabitants). Incidence rates are shown for each age group. B) Frequency of symptoms reported in OROV-positive patients. C) Boxplots of Ct values from OROV reverse transcription PCR in patients with or without fever, headache, myalgia, and retroorbital pain. Black horizontal bars indicate medians, tops and bottoms of boxes indicate interquartile ranges, vertical whiskers indicate ranges. Gray dots represent individual Ct values. p values determined by 2-sided Mann–Whitney U test for each comparison.

We observed a marked predominance of male patients in OROV cases (Figure 2, panel A); male-to-female ratio was 1.5:1. Most cases occurred in persons >20 years of age, suggesting that adult men may be disproportionately affected by this outbreak. The most frequently reported symptoms were fever (307/339 [90.56%]), headache (275/339 [81.12%]), myalgia (232/339 [68.44%]), and retroorbital pain (113/339 [33.33%]) (Figure 2, panel B). We observed no significant differences in symptoms between male and female patients. We used real-time RT-PCR Ct values as a proxy for the patient’s plasmatic viral load to investigate its relationship to symptomatology. Patients with fever exhibited significantly lower median Ct values than those without fever (p = 0.017), suggesting a higher viral load in febrile patients. We observed a similar trend for headache (p = 0.043), myalgia (p = 0.025), and retroorbital pain (p = 0.017) (Figure 2, panel C). Comparisons for other symptoms did not yield statistically significant differences (Appendix Figure 1).

**Figure 3:**
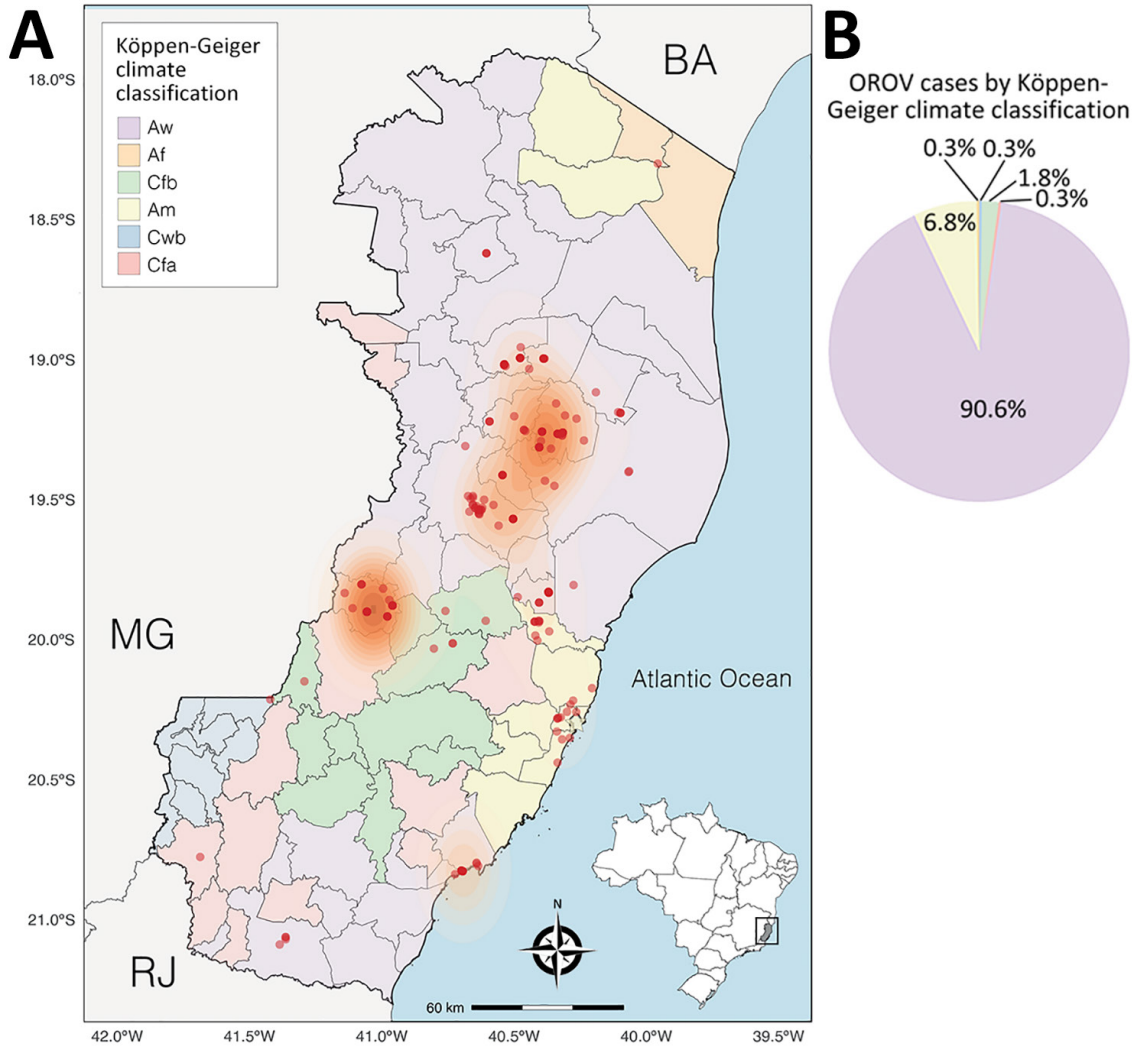
Spatial distribution of Oropouche virus cases and climate classification in Espírito Santo, Brazil. A) Espírito Santo, with municipalities colored according to the Köppen-Geiger climate classification. Red dots indicate cases. Orange shading represents case density. Inset shows location of Espírito Santo state in Brazil. B) Pie chart showing the percentage of cases in each Köppen-Geiger climate category.

During the OROV outbreak in Espírito Santo, the virus spread across 17 municipalities, culminating in 8.84 cases/100,000 inhabitants statewide. Most cases were concentrated in 2 distinct hotspots: the regions surrounding the municipalities of Colatina/Rio Bananal and Laranja da Terra (Figure 3, panel A). In total, 332/339 (98%) of the diagnosed cases were associated with tropical climates, in accordance with Köppen-Geiger climate classification; 308 (≈91%) occurred in municipalities classified as having a tropical savanna climate (Köppen-Geiger classification Aw), and 24 (7%) were linked to tropical monsoon climate (Köppen-Geiger classification Am) (Figure 3, panel B). In contrast, only 7/339 cases (≈2%) were distributed across municipalities with temperate climates, such as humid subtropical (Köppen-Geiger classification Cfa) and temperate oceanic (Köppen-Geiger classification Cfb). No cases were reported in areas classified as subtropical highland climate (Köppen-Geiger classification Cwb).

**Figure 4:**
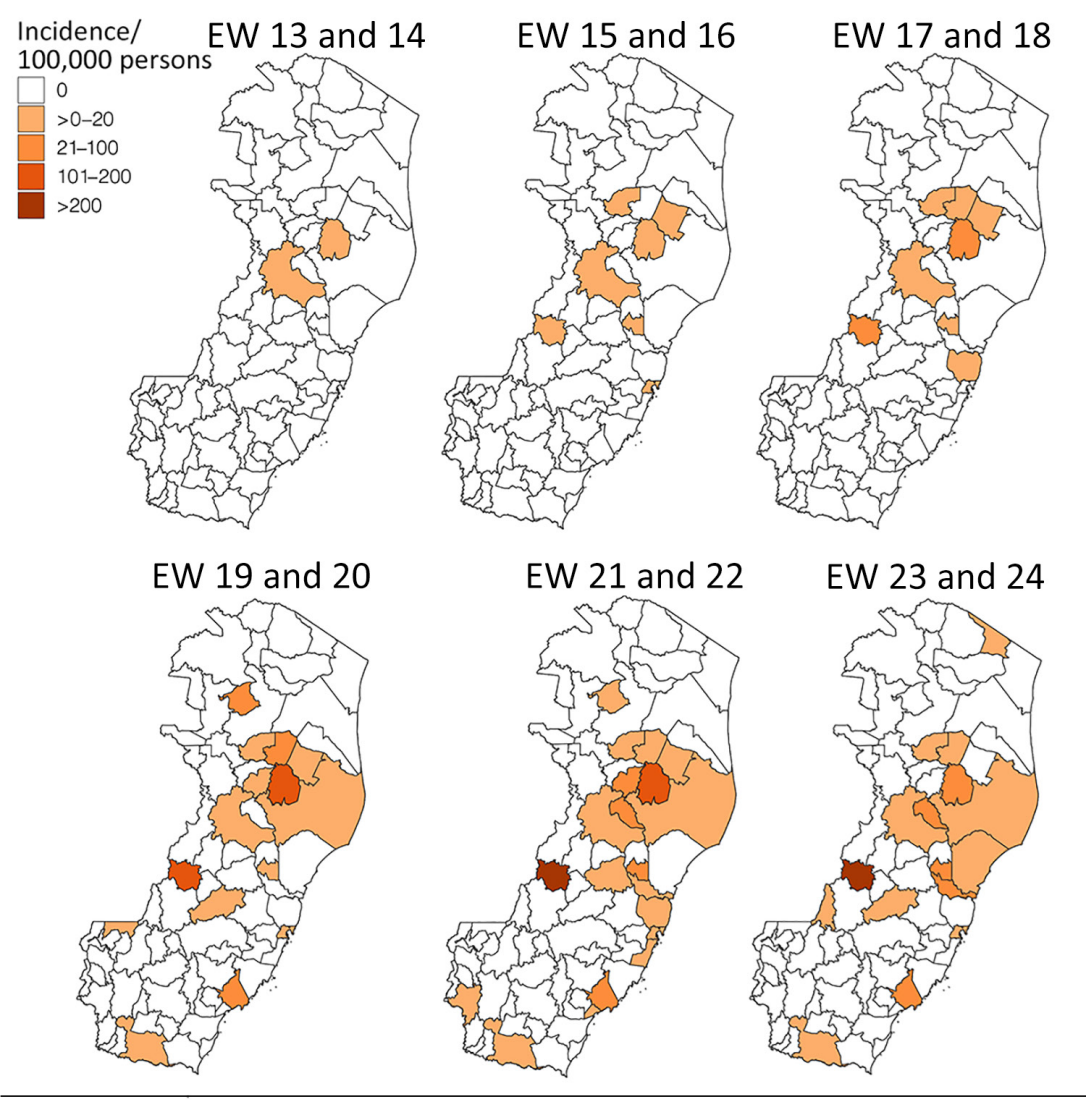
Oropouche virus case incidence by municipality and 2-week epidemiologic period, Espírito Santo state, Brazil. EW, epidemiologic week.

The initial spread of OROV in Espírito Santo followed a clear spatial pattern, predominantly affecting municipalities within the tropical savanna climate (Figure 4). This dissemination phase, epidemiologic weeks 17–18, marked the entry of the virus into areas with different climatic conditions. The peak incidence, exceeding 200 cases/100,000 inhabitants, was recorded at the municipality level during epidemiologic weeks 21–24, particularly in the epicentral municipalities of Colatina and Laranja da Terra. Those areas, situated within the tropical savanna climate zone, appear to have served as primary foci for transmitting OROV throughout the state. To characterize the ecologic niches contributing to the introduction and spread of OROV in Espírito Santo, we examined the association between OROV prevalence and the cultivated areas for the 10 most widely grown crops in the state. We found significant associations between specific crops and OROV incidence (Table); robusta coffee (Spearman correlation coefficient [ρ] = 0.55, p = 0.004), cacao (ρ = 0.54, p = 0.005), coconut (ρ = 0.43, p = 0.003), and pepper (ρ = 0.43, p = 0.034) displayed moderate positive correlations with the number of OROV cases.

**Table:** Association between agricultural crop cultivation and Oropouche virus cases, Espírito Santo, Brazil*

Crop	Spearman ρ (95% CI)	p value
Robusta coffee	0.549 (0.185–0.781)	**0.004**
Cocoa	0.530 (0.159–0.770)	**0.006**
Coconut	0.434 (0.035–0.714)	**0.030**
Pepper	0.426 (0.024–0.709)	**0.034**
Banana	0.345 (−0.070 to 0.658)	0.091
Orange	0.037 (−0.374 to 0.436)	0.861
Cassava	0.026 (−0.384 to 0.427)	0.902
Corn	−0.086 (−0.475 to 0.331)	0.684
Bean	−0.213 (−0.569 to 0.211)	0.307
Arabica coffee	−0.259 (−0.601 to 0.164)	0.212

*Bold text indicates statistically significant correlation (p<0.05).

**Figure 5:**
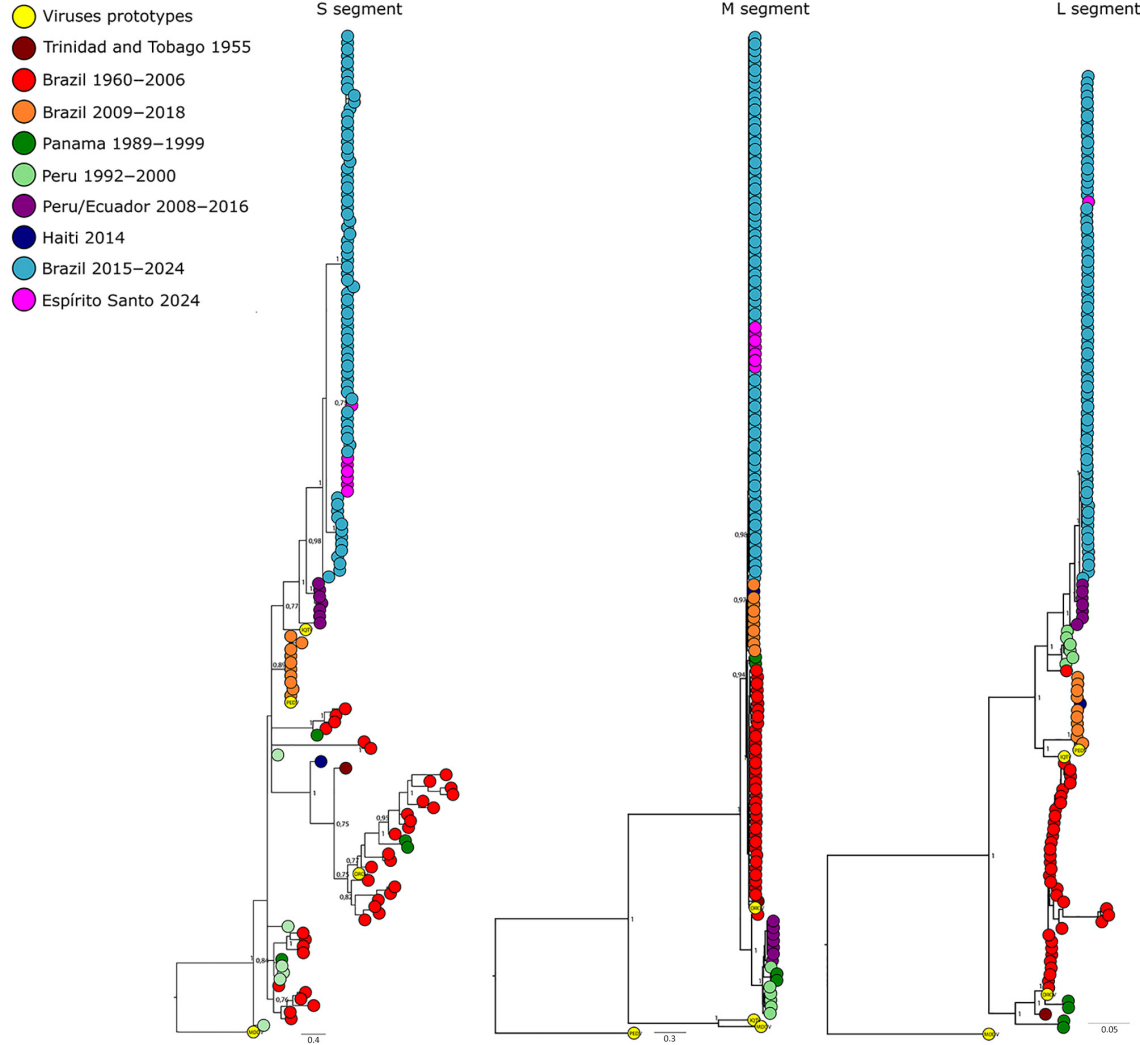
Bayesian phylogenetic trees created from analysis of Oropouche virus segments associated with the reassortant lineage M_1_L_2_S_2_ in Espírito Santo, Brazil, and reference sequences. Each tree includes a representative subsample of genomes from the epidemic clade (highlighted); tip colors indicate sampling locations. Branch support values are shown for the main clades. Scale bars indicate the number of nucleotide substitutions per site. S, small; M, medium; L, long.

Phylogenetic analysis of each genomic segment individually showed that all cases detected within Espírito Santo belong to the novel reassortant M_1_L_2_S_2_ (OROV_BR2015–2024_) lineage (*15*) with high branch support (posterior probability >0.98). That lineage circulated in the Amazon Basin during 2023–2024, causing a major outbreak (Figure 5). Of interest, in the S and M trees, the Espírito Santo samples do not form a monophyletic clade, which indicated multiple introductions of OROV into the state.

## Discussion

The emergence and rapid spread of arboviruses beyond their traditional endemic regions is driven by changing climate patterns and human land use (*7*). Our study used high-resolution data to document the unprecedented establishment of OROV in Brazil’s Atlantic Forest, showing its adaptations and spread beyond the Amazon Basin. The expansion mirrors patterns observed for other arboviruses, such as ZIKV (*36*), CHIKV (*37*), and West Nile virus (*38*), in which changes in vector ecology, human mobility, and environmental conditions have led to emergence of those viruses in previously unaffected regions (*6*). OROV’s successful establishment in southeast Brazil underscores both an immediate public health concern and the complex ecologic and epidemiologic factors enabling arboviral adaptation to novel environments.

Our findings revealed a marked epidemiologic shift with the emergence and rapid establishment of OROV transmission in the state amidst the ongoing endemic circulation of DENV and CHIKV. Within ≈11 weeks from its initial detection, OROV reached infection levels comparable to those of endemic arboviruses in Espírito Santo, suggesting robust viral transmission. The concurrent presence of OROV alongside DENV and CHIKV, without evident competitive interference, could reflect vector-specific ecologic differences: although DENV and CHIKV are transmitted predominantly by *Aedes* spp. mosquitoes in urban areas (*39*), OROV transmission is driven mainly by *C. paraensis* midges (*3*,*27*). The estimated transmissibility, with R_t_ peaking at ≈3.0, parallels the dynamics seen in both urban and sylvatic arboviruses within immunologically naive or mixed populations. Similar patterns were reported for ZIKV; R_t_ values were 2.6–4.8 in French Polynesia (*40*), 3.0–6.6 in Colombia (*41*), and as high as 2.5 in Brazil (*30*). Likewise, MAYV, endemic to the Amazon region, showed R_t_ values of 2.1–2.9 within its primary range, decreasing to 1.1–1.3 in non-Amazon regions (*31*). The high transmissibility of OROV observed in Espírito Santo could be attributed to multiple factors, including the predominantly mild or asymptomatic nature of OROV infections (*3*,*27*) and favorable local conditions for *C. paraensis* midge proliferation (*28*). Our R_t_ estimates might underestimate transmissibility by excluding unreported asymptomatic and mild cases. However, if underreporting remains stable, the inferred trends should still reliably reflect epidemic dynamics (*32*).

The demographic profile of affected persons in this outbreak showed predominance of adult male case-patients, which contrasts with previous OROV outbreaks (*12*,*14*). The male predominance may reflect occupational exposure in rural agricultural activities, increasing contact with *C. paraensis* vectors, as seen in previous Brazil outbreaks where farming was a major risk factor (*42*). Clinical manifestations aligned with previous OROV cases across diverse regions, including recent Amazon outbreaks (*12*,*14*,*43*). The association between key symptoms and lower Ct values suggested that higher viral loads may drive the intensity of clinical manifestations, as documented for other arboviruses, such as DENV (*44*,*45*), yet remains largely unexplored for OROV. Of note, recent fatal OROV cases in nonendemic regions of Brazil, characterized by low Ct values and rapid progression to severe hemorrhagic symptoms within days, underscored the potential link between viral load and disease severity (*18*).

Phylogenetic analysis of OROV genomic sequences from Espírito Santo revealed a complex pattern of viral spread, with multiple independent introductions of the virus into the state. The samples clustered within the OROV_Brazil2015–2024_ clade, specifically within the M_1_L_2_S_2_ reassortant lineage that caused the recent outbreak in the Amazon region (*14*). The absence of monophyly in the S and M segment trees suggests that multiple introduction events occurred followed by local spread through different routes, as described in Gräf et al. (*17*). That molecular pattern corroborates the epidemiologic data showing the initial emergence of imported cases followed by rapid establishment of autochthonous transmission in different municipalities. Further genomic surveillance is needed to fully understand the dispersal patterns and the state’s role in the virus’s spread to other regions, highlighting the need for comprehensive and continuous surveillance throughout the region.

The predominance of OROV transmission in regions with tropical climates (Köppen-Geiger classifications Aw and Am) highlights the critical role of environmental conditions that favor a high prevalence of potential vectors, particularly *C. paraensis* midges. Although comprehensive studies on *Culicoides* spp. distribution across climate zones of Brazil are lacking, studies in Europe have documented strong associations between Köppen climate classifications and *Culicoides* spp. diversity (*46*). The tropical climates of Espírito Santo outbreak zones have high temperatures and seasonal rainfall, likely creating optimal conditions for *C. paraensis* breeding. Those environments provide abundant organic matter for larval development; decomposing vegetation, fruit waste, banana tree stumps, and cacao husks, common agricultural byproducts in the region, are ideal breeding sites (*3*,*4*,*27*). Indeed, studies report peak *C. paraensis* midge populations during the rainy season; temperatures are 30°C–32°C and relative humidity 75%–85% (*47*), favorable macroclimatic and microclimatic conditions for vector proliferation. In contrast, temperate and subtropical highland climates, characterized by lower temperatures, more evenly distributed rainfall, and distinct vegetation, present less favorable conditions for vector establishment. Despite the widespread distribution of *Culicoides* spp. midges across the Americas (*28*), no cases of OROV had been reported in Espírito Santo state until 2024. The introduction of OROV into Espírito Santo is estimated to have occurred multiple times during February–March 2024 (*17*), and its establishment may reflect ecologic shifts that have amplified vector density or increased human–vector contact, particularly among susceptible populations in Espírito Santo and other regions of Brazil. Espírito Santo’s recent biting midge infestation in 2019 (*48*) likely supported conditions for OROV emergence by increasing vector populations in previously unaffected areas. Climate change projections in Europe suggest a shift toward warmer and wetter conditions that may transform humid subtropical climates into subtropical climates with hot summers, further promoting *Culicoides* spp. establishment (*46*). Modeling studies indicate that climate-driven increases in temperature and extreme weather events could expand arboviral transmission in tropical regions like Espírito Santo, as observed for DENV (*49*). These ecologic changes likely interact with agricultural and land-use patterns, shaping OROV transmission and creating corridors that intensify virus-vector-host interactions. Further research is warranted to quantify the relationships between climatic variables, *C. paraensis* species dynamics, and OROV transmission in Espírito Santo and other countries.

The significant correlations between OROV incidence and specific crops in Espírito Santo underscore the complex interactions between agricultural landscapes and arboviral transmission dynamics. Outside the Amazon region, OROV transmission has been predominantly associated with rural settings, where *C. paraensis* midges find optimal breeding conditions. *C. paraensis* larvae develop effectively in microhabitats created by decaying organic matter from banana and cacao plantations (*3*,*4*,*27*); of note, recent outbreaks outside the Amazon have occurred along the Atlantic Forest biome where these crops are prevalent (*17*). The emergence of OROV in Espírito Santo’s regions with high densities of robusta coffee, pepper, and coconut cultivation represents a novel association that warrants further investigation, because it would broaden our understanding of potential agricultural landscapes that may support *C. paraensis* populations. That pattern aligns with the vector’s documented ability to occupy both sylvatic and anthropic environments (*28*) and suggests that agricultural expansion may create ecologic pathways that encourage viral spread. The fragmentation of forest landscapes by agricultural activities likely intensifies human–vector contact, particularly in areas where multiple crops create diverse microhabitats suitable for vector breeding. The strong presence of OROV in Espírito Santo’s major agricultural regions, especially where coffee and cacao cultivation predominate, emphasizes the need for detailed ecologic studies of *C. paraensis* midges in those settings to identify and predict potential outbreak hotspots.

Our study provides valuable insights into environmental and ecologic factors influencing OROV emergence in Espírito Santo. A limitation is that inferences about spatial dissemination relied on assumptions regarding the primary vector, *C. paraensis* midges, without directly assessing its competence, density, or dispersal potential, which may affect estimates of transmission dynamics. In addition, reliance on secondary epidemiologic data, rather than targeted field sampling, limits the granularity and completeness of information on transmission patterns and hotspots. Observed correlations between OROV cases and crops might also reflect geographic clustering effects, potentially introducing spatial biases. Future research with fine-scale spatial modeling and systematic vector surveillance is essential to clarify interactions among environmental variables, vector ecology, and OROV transmission dynamics.

In conclusion, our study reveals a substantial epidemiologic shift marked by the emergence and establishment of OROV in Espírito Santo, underscoring the virus’s capacity to adapt to new ecologic landscapes outside its Amazon origins. The rapid rise in OROV cases to levels comparable with established arboviruses like DENV and CHIKV, without apparent competitive inhibition, highlights the distinct ecologic niches exploited by *Culicoides* spp. vectors in periurban and rural areas. The interplay between tropical climate, expanding agricultural landscapes, and favorable breeding sites for *C. paraensis* midges likely enabled this outbreak, positioning Espírito Santo as a potential hotspot for arboviral transmission amid environmental and climatic changes. As such, our findings call for heightened surveillance of both human cases and vector populations along with further investigation into the eco-epidemiologic drivers of OROV in Atlantic Forest regions. Understanding those dynamics will be crucial to predict, prevent, and respond to future outbreaks of OROV and other emerging arboviruses in similar environments.

Appendix Additional information about Oropouche virus outbreak in Espírito Santo state, Brazil, 2024.
