# Supplementary material for: Emergence of Oropouche Virus in Espírito Santo State, Brazil, 2024
Source: Emerg Infect Dis. 2025 Jun;31(6):1178–88. doi: 10.3201/eid3106.241946 (PMC12123935; doi:10.3201/eid3106.241946)
Supplement: Appendix — Additional information about Oropouche virus outbreak in Espírito Santo state, Brazil, 2024. [file 24-1946-Techapp-s1.pdf]

Article DOI: https://doi.org/10.3201/eid3106.241946

EID cannot ensure accessibility for supplementary materials supplied by authors. Readers who have difficulty accessing supplementary content should contact the authors for assistance.

Emergence of Oropouche Virus in Espírito Santo State, Brazil, 2024

Appendix

Supplemental Methods for Estimation of Generation Time and Calculation of the Time-Varying Reproduction Number

Natural History Parameter Estimates

Human-to-Mosquito Generation Time

The human-to-mosquito generation time represents the interval between human infection and the mosquito's ability to transmit the virus through an infectious blood meal. This period encompasses the intrinsic incubation period (from infection to symptom onset) and the duration from symptom onset to viral clearance.

Intrinsic Incubation Period

While not precisely determined, the incubation period of Oropouche fever is estimated to range from 4 to 8 days in natural infections. We assumed a log-normal distribution based on Lessler et al.'s approach (*1*). Using the Metropolis-Hastings Markov chain Monte Carlo (MCMC) algorithm, we calibrated the model to estimate the viral incubation characteristics.

We estimated a mean incubation period μ_{IP} of 6.0 (95% CrI: 5.8–6.3) days and a standard deviation σ_{IP} of 1.2 (95% CrI: 1.0–1.5) days.

Time to Viral Clearance

The time to viral clearance refers to the duration from the onset of symptoms until the virus is no longer detectable in the blood. This period represents the window during which the virus is present in the bloodstream and may potentially be transmissible to mosquito vectors in the case of arboviruses. For OROV, observational data in humans accounts that the viral

clearance period typically lasts 1-5 days, with decreasing probabilities of viral detections as the days advance (*2*).

Following established methods for other arboviruses (*3*,*4*), we assumed that OROV viral clearance follows a Gamma distribution with shape parameter $\boldsymbol{\alpha_C}$ and scale parameter $\boldsymbol{\beta_C}$, estimated using the Metropolis-Hastings MCMC algorithm (Appendix Figure 1). In Appendix Table 1, we report the mean ($\boldsymbol{\mu_C = \alpha_C \beta_C}$) and standard deviation ($\boldsymbol{\sigma_C = \alpha_C \beta_C^2}$) of the time to viral clearance.

We modeled infectiousness similar to those used for Zika (*3*) and Mayaro (*4*) virus infections, with transmission beginning 1.5 days before symptom onset and ending 1.5-2 days before viral detection becomes undetectable.

By linearly scaling the time dependence by a factor $\boldsymbol{s = (\mu_{IP} - 1.5)/\mu_{IP}}$, we derived the OROV human generation time, calculating its mean mean $\boldsymbol{\mu_h = s(\mu_{IP} + \mu_C)}$ and standard deviation $\boldsymbol{\sigma_h = s\sqrt{\sigma_{IP}^2 + \sigma_C^2}}$ to characterize the virus's transmission dynamics.

Extrinsic Incubation Period

We identified a single peer-reviewed article that investigated the susceptibility of North American mosquitoes and midges to OROV (*5*). This study provided sufficient information on the number of mosquitoes tested at each day post-infection, which is critical for estimating the extrinsic incubation period (EIP). The research evaluated the susceptibility of *Culex quinquefasciatus* and *Culicoides sonorensis*. Given the limited data, we aggregated information across all insect species and used it as a proxy for the susceptibility of *Culicoides paraensis*. The EIP was defined as the interval between infection and the dissemination of OROV to the salivary glands, rendering the mosquito capable of transmitting the virus.

We assumed that the extrinsic incubation period is Gamma distributed with shape parameter k_{EIP} and scale parameter θ_{EIP}, following previous studies (*3*,*4*). Using a Binomial likelihood function to estimate the probability that a mosquito is infectious by day *t*, we obtain mean posterior estimates of k_{EIP} = 10.1 (95% CrI: 6.5–14.3) and θ_{EIP} = 1.21 (95% CrI: 0.8–1.7). This results in a mean EIP of 12.2 (95% CrI: 5.2–24.3) days with a standard deviation of 3.8 (95% CrI: 2.0–6.4) days (Appendix Table 1). Appendix Figure 2 shows the fitted probability density function, cumulative distribution function, and observed data.

Mosquito-to-Human Generation Time

We followed the methodology proposed by Fergunson et al. (*3*) and Caicedo et al. (*4*) to estimate the mosquito-to-human generation time, defined as the time between a vector being infected and subsequently infecting a human. However, considering the longer life cycle of *Culicoides* spp. compared to mosquitoes, we adjusted the parameters of the Gamma distribution to reflect the typical lifespan of the biting midge (*6*). The mosquito-to-human generation time was estimated from the estimated EIP and the mosquito daily mortality rate. We assume that the mosquito mortality rate ε is Gamma-distributed with a mean of 0.07/day and a standard deviation of 0.01/day. We estimate the mean μ_m and standard deviation σ_m of the mosquito-to-human generation time numerically, i.e. sampling the shape parameter k_{EIP} and scale parameter θ_{EIP} from their posterior distributions and ε from the Gamma distribution with mean of 0.07/day and standard deviation of 0.01/day.

We estimated a mean mosquito-to-human generation time of 11.8 (95% CrI: 5.4–20.9) days and a standard deviation of 4.1 (95% CrI: 3.3–4.9) days (Appendix Table 1).

Generation Time of OROV

Combining the estimates of the human-to-mosquito generation time with those of the mosquito-to-human generation time, we estimate that the distribution of the generation time of OROV (i.e. the time between infection of a human case and infection of the secondary human cases that case causes) has a mean of 18.0 (95% CrI: 10.7–28.6) days and a standard deviation of 4.3 (95% CrI: 3.4–5.1) days (Appendix Table 1).

Estimates of the Reproduction Number, *R*

We estimated the instantaneous reproduction number *R* for the 2024 OROV outbreak in Espírito Santo, Brazil using the daily case counts and the generation time distribution estimated in the previous section.

The instantaneous reproduction number (*R*) was estimated using the EpiEstim package (*7*) in R version 4.4.1 (R Foundation for Statistical Computing, https://www.R-project.org) within 1-week sliding time windows. For each window, *R* was computed as the median of the weekly instantaneous reproduction numbers, weighted by the weekly incidence. The resulting *R* estimates were plotted at the midpoint of their respective time windows. The estimation process employed the uncertain_si method from the estimate_R() function, incorporating a prior

distribution for *R* with a mean and standard deviation of 5, as well as the mean and standard deviation of the generation time described in Appendix Table 2.

The uncertain_si approach accounts for uncertainty in the serial interval distribution, as outlined by Cori et al. (*7*). Specifically, the mean (μ) and standard deviation (σ) of the serial interval were allowed to vary within truncated normal distributions, parameterized as shown in Appendix Table 2. From these distributions, *n1* pairs of μ and σ were sampled, and for each pair, *n2* samples were drawn from the posterior distribution of *R* for each time window. Conditional on the serial interval distribution obtained, this process generated a pooled sample of size $n1 \times n2$, representing the joint posterior distribution of *R* across all time windows.

References

1. Lessler J, Ott CT, Carcelen AC, Konikoff JM, Williamson J, Bi Q, et al. Times to key events in Zika virus infection and implications for blood donation: a systematic review. Bull World Health Organ. 2016;94:841–9. PubMed https://doi.org/10.2471/BLT.16.174540

2. Pinheiro FP, Travassos da Rosa AP, Travassos da Rosa JF, Ishak R, Freitas RB, Gomes ML, et al. Oropouche virus. Am J Trop Med Hyg. 1981;30:149–60. PubMed https://doi.org/10.4269/ajtmh.1981.30.149

3. Ferguson NM, Cucunubá ZM, Dorigatti I, Nedjati-Gilani GL, Donnelly CA, Basáñez MG, et al. Countering the Zika epidemic in Latin America. Science. 2016;353:353–4. PubMed https://doi.org/10.1126/science.aag0219

4. Caicedo EY, Charniga K, Rueda A, Dorigatti I, Mendez Y, Hamlet A, et al. The epidemiology of Mayaro virus in the Americas: a systematic review and key parameter estimates for outbreak modelling. PLoS Negl Trop Dis. 2021;15:e0009418. PubMed https://doi.org/10.1371/journal.pntd.0009418

5. McGregor BL, Connelly CR, Kenney JL. Infection, dissemination, and transmission potential of North American *Culex quinquefasciatus, Culex tarsalis*, and *Culicoides sonorensis* for Oropouche virus. Viruses. 2021;13:226. PubMed https://doi.org/10.3390/v13020226

6. Mellor PS, Boorman J, Baylis M. *Culicoides* biting midges: their role as arbovirus vectors. Annu Rev Entomol. 2000;45:307–40. PubMed https://doi.org/10.1146/annurev.ento.45.1.307

7. Bhatia S, Wardle J, Nash RK, Nouvellet P, Cori A. Extending EpiEstim to estimate the transmission advantage of pathogen variants in real-time: SARS-CoV-2 as a case-study. Epidemics. 2023;44:100692. PubMed https://doi.org/10.1016/j.epidem.2023.100692

Appendix Table 1. Estimates of the mean and standard deviation of the generation time distribution and its components for Oropouche virus in Espírito Santo State, Brazil, 2024*

Estimate	Mean (95% CrI)	Standard deviation (95% CrI)	Source†
Intrinsic incubation period, d	6.0 (5.8–6.3)	1.2 (1.0–1.5)	Estimated with data from (2)
Time to viral clearance, d	2.3 (1.3–3.9)	1.3 (0.6–2.9)	Estimated with data from (2)
Human-to-mosquito generation time, d	6.2 (5.3–7.7)	1.3 (0.9–2.5)	Estimated
Extrinsic incubation period, d	12.2 (6.5 - 14.3)	1.2 (0.8–1.7)	Estimated with data from (*5*)
Vector lifetime, d (*Culicoides* spp.)	15 (fixed)	2.5 (fixed)	From (*6*)
Mosquito-to-human generation time, d	11.8 (5.4–20.9)	4.1 (3.2–4.9)	Estimated
Oropouche virus generation time, d	18.0 (10.7–28.6)	4.3 (3.4–5.1)	Estimated

*CrI, credible interval.
†From Appendix references.

Appendix Table 2. Values used in estimate_R() function in EpiEstim package

Parameter	Days
mean_si	18.0
std_mean_si	1.0
min_mean_si	10.7
max_mean_si	28.6
std_si	4.3
std_std_si	0.43
min_std_si	3.4
max_std_si	5.1

Appendix Table 3. Oropouche samples and metadata used in study of Oropouche virus outbreak in Espírito Santo State, Brazil, 2024

Accession number ID	Genome segment	Collection date	Location	Major groups classification
KP026179.1	Long	1955–00–00	Trinidad and Tobago_SangreGrande	TT_1955
KP026180.1	Medium	1955–00–00	Trinidad and Tobago_SangreGrande	TT_1955
KP026181.1	Small	1955–00–00	Trinidad and Tobago_SangreGrande	TT_1955
KP052850.1	Long	1960–00–00	Brazil	OROV prototype
KP052851.1	Medium	1960–00–00	Brazil	OROV prototype
KP052852.1	Small	1960–00–00	Brazil	OROV prototype
MG747524.1	Small	1960–00–00	Brazil	BR_1960–2006
MG747525.1	Medium	1960–00–00	Brazil	BR_1960–2006
MG747526.1	Long	1960–00–00	Brazil	BR_1960–2006
MG747527.1	Small	1961–00–00	Brazil	BR_1960–2006
MG747528.1	Medium	1961–00–00	Brazil	BR_1960–2006
MG747529.1	Long	1961–00–00	Brazil	BR_1960–2006
MG747530.1	Small	1961–00–00	Brazil	BR_1960–2006
MG747531.1	Medium	1961–00–00	Brazil	BR_1960–2006
MG747532.1	Long	1961–00–00	Brazil	BR_1960–2006
MG747533.1	Small	1967–00–00	Brazil	BR_1960–2006
MG747534.1	Medium	1967–00–00	Brazil	BR_1960–2006
MG747535.1	Long	1967–00–00	Brazil	BR_1960–2006
MG747536.1	Small	1968–00–00	Brazil	BR_1960–2006
MG747537.1	Medium	1968–00–00	Brazil	BR_1960–2006
MG747538.1	Long	1968–00–00	Brazil	BR_1960–2006
MG747539.1	Small	1971–00–00	Brazil	BR_1960–2006
MG747540.1	Medium	1971–00–00	Brazil	BR_1960–2006
MG747541.1	Long	1971–00–00	Brazil	BR_1960–2006
MG747542.1	Small	1971–00–00	Brazil	BR_1960–2006
MG747543.1	Medium	1971–00–00	Brazil	BR_1960–2006
MG747544.1	Long	1971–00–00	Brazil	BR_1960–2006
MG747545.1	Small	1971–00–00	Brazil	BR_1960–2006
MG747546.1	Medium	1971–00–00	Brazil	BR_1960–2006

Accession number ID	Genome segment	Collection date	Location	Major groups classification
MG747547.1	Long	1971–00–00	Brazil	BR_1960–2006
MG747548.1	Small	1978–00–00	Brazil	BR_1960–2006
MG747549.1	Medium	1978–00–00	Brazil	BR_1960–2006
MG747550.1	Long	1978–00–00	Brazil	BR_1960–2006
MG747551.1	Small	1979–00–00	Brazil	BR_1960–2006
MG747552.1	Medium	1979–00–00	Brazil	BR_1960–2006
MG747553.1	Long	1979–00–00	Brazil	BR_1960–2006
MG747506.1	Small	1980–00–00	Brazil	BR_1960–2006
MG747507.1	Medium	1980–00–00	Brazil	BR_1960–2006
MG747508.1	Long	1980–00–00	Brazil	BR_1960–2006
MG747509.1	Small	1980–00–00	Brazil	BR_1960–2006
MG747510.1	Medium	1980–00–00	Brazil	BR_1960–2006
MG747511.1	Long	1980–00–00	Brazil	BR_1960–2006
MG747554.1	Small	1980–00–00	Brazil	BR_1960–2006
MG747555.1	Medium	1980–00–00	Brazil	BR_1960–2006
MG747556.1	Long	1980–00–00	Brazil	BR_1960–2006
MG747512.1	Small	1988–00–00	Brazil	BR_1960–2006
MG747513.1	Medium	1988–00–00	Brazil	BR_1960–2006
MG747514.1	Long	1988–00–00	Brazil	BR_1960–2006
MG747515.1	Small	1988–00–00	Brazil	BR_1960–2006
MG747516.1	Medium	1988–00–00	Brazil	BR_1960–2006
MG747517.1	Long	1988–00–00	Brazil	BR_1960–2006
KP795075.1	Long	1989–00–00	Panama_SanMiguelito	Panama_1989–1999
KP795076.1	Medium	1989–00–00	Panama_SanMiguelito	Panama_1989–1999
KP795077.1	Small	1989–00–00	Panama_SanMiguelito	Panama_1989–1999
KP795081.1	Long	1989–00–00	Panama_CidadedoPanama	Panama_1989–1999
KP795082.1	Medium	1989–00–00	Panama_CidadedoPanama	Panama_1989–1999
KP795083.1	Small	1989–00–00	Panama_CidadedoPanama	Panama_1989–1999
KP795078.1	Long	1989–08–00	Panama	Panama_1989–1999
KP795079.1	Medium	1989–08–00	Panama	Panama_1989–1999
KP795080.1	Small	1989–08–00	Panama	Panama_1989–1999
MG747602.1	Small	1990–00–00	Brazil	BR_1960–2006
MG747603.1	Medium	1990–00–00	Brazil	BR_1960–2006
MG747604.1	Long	1990–00–00	Brazil	BR_1960–2006
MG747605.1	Small	1991–00–00	Brazil	BR_1960–2006
MG747606.1	Medium	1991–00–00	Brazil	BR_1960–2006
MG747607.1	Long	1991–00–00	Brazil	BR_1960–2006
PP357048.1	Long	1991–00–00	Brazil_Rondonia	BR_1960–2006
PP357049.1	Medium	1991–00–00	Brazil_Rondonia	BR_1960–2006
PP357050.1	Small	1991–00–00	Brazil_Rondonia	BR_1960–2006
KP795072.1	Long	1992–04–00	Peru	Peru_1992–2000
KP795073.1	Medium	1992–04–00	Peru	Peru_1992–2000
KP795074.1	Small	1992–04–00	Peru	Peru_1992–2000
MG747518.1	Small	1993–00–00	Brazil	BR_1960–2006
MG747519.1	Medium	1993–00–00	Brazil	BR_1960–2006
MG747520.1	Long	1993–00–00	Brazil	BR_1960–2006
MG747557.1	Small	1994–00–00	Brazil	BR_1960–2006
MG747558.1	Medium	1994–00–00	Brazil	BR_1960–2006
MG747559.1	Long	1994–00–00	Brazil	BR_1960–2006
MG747560.1	Small	1994–00–00	Brazil	BR_1960–2006
MG747561.1	Medium	1994–00–00	Brazil	BR_1960–2006
MG747562.1	Long	1994–00–00	Brazil	BR_1960–2006
MG747563.1	Small	1994–00–00	Brazil	BR_1960–2006
MG747564.1	Medium	1994–00–00	Brazil	BR_1960–2006
MG747565.1	Long	1994–00–00	Brazil	BR_1960–2006
MG747566.1	Small	1994–00–00	Brazil	BR_1960–2006
MG747567.1	Medium	1994–00–00	Brazil	BR_1960–2006
MG747568.1	Long	1994–00–00	Brazil	BR_1960–2006
MG747569.1	Small	1994–00–00	Brazil	BR_1960–2006
MG747570.1	Medium	1994–00–00	Brazil	BR_1960–2006
MG747571.1	Long	1994–00–00	Brazil	BR_1960–2006
KP795096.1	Long	1994–02–00	Peru	Peru_1992–2000
KP795097.1	Medium	1994–02–00	Peru	Peru_1992–2000
KP795098.1	Small	1994–02–00	Peru	Peru_1992–2000
KP795087.1	Long	1995–06–00	Peru	Peru_1992–2000
KP795088.1	Medium	1995–06–00	Peru	Peru_1992–2000
KP795089.1	Small	1995–06–00	Peru	Peru_1992–2000
MG747503.1	Small	1996–00–00	Brazil	BR_1960–2006

Accession number ID	Genome segment	Collection date	Location	Major groups classification
MG747504.1	Medium	1996–00–00	Brazil	BR_1960–2006
MG747505.1	Long	1996–00–00	Brazil	BR_1960–2006
MG747572.1	Small	1996–00–00	Brazil	BR_1960–2006
MG747573.1	Medium	1996–00–00	Brazil	BR_1960–2006
MG747574.1	Long	1996–00–00	Brazil	BR_1960–2006
MG747575.1	Small	1996–00–00	Brazil	BR_1960–2006
MG747576.1	Medium	1996–00–00	Brazil	BR_1960–2006
MG747577.1	Long	1996–00–00	Brazil	BR_1960–2006
MG747578.1	Small	1996–00–00	Brazil	BR_1960–2006
MG747579.1	Medium	1996–00–00	Brazil	BR_1960–2006
MG747580.1	Long	1996–00–00	Brazil	BR_1960–2006
KP795090.1	Long	1997–08–00	Peru	Peru_1992–2000
KP795091.1	Medium	1997–08–00	Peru	Peru_1992–2000
KP795092.1	Small	1997–08–00	Peru	Peru_1992–2000
KP795093.1	Long	1998–04–00	Peru	Peru_1992–2000
KP795094.1	Medium	1998–04–00	Peru	Peru_1992–2000
KP795095.1	Small	1998–04–00	Peru	Peru_1992–2000
KF697142.1	Long	1999–00–00	Peru_Iquitos	IQTV prototype
KF697143.1	Medium	1999–00–00	Peru_Iquitos	IQTV prototype
KF697144.1	Small	1999–00–00	Peru_Iquitos	IQTV prototype
KP795102.1	Long	1999–10–00	Panama	Panama_1989–1999
KP795103.1	Medium	1999–10–00	Panama	Panama_1989–1999
KP795104.1	Small	1999–10–00	Panama	Panama_1989–1999
MG747521.1	Small	2000–00–00	Brazil	BR_1960–2006
MG747522.1	Medium	2000–00–00	Brazil	BR_1960–2006
MG747523.1	Long	2000–00–00	Brazil	BR_1960–2006
KP795099.1	Long	2000–05–00	Peru	Peru_1992–2000
KP795100.1	Medium	2000–05–00	Peru	Peru_1992–2000
KP795101.1	Small	2000–05–00	Peru	Peru_1992–2000
MG747581.1	Small	2003–00–00	Brazil	BR_1960–2006
MG747582.1	Medium	2003–00–00	Brazil	BR_1960–2006
MG747583.1	Long	2003–00–00	Brazil	BR_1960–2006
MG747584.1	Small	2003–00–00	Brazil	BR_1960–2006
MG747585.1	Medium	2003–00–00	Brazil	BR_1960–2006
MG747586.1	Long	2003–00–00	Brazil	BR_1960–2006
MG747587.1	Small	2004–00–00	Brazil	BR_1960–2006
MG747588.1	Medium	2004–00–00	Brazil	BR_1960–2006
MG747589.1	Long	2004–00–00	Brazil	BR_1960–2006
MG747590.1	Small	2004–00–00	Brazil	BR_1960–2006
MG747591.1	Medium	2004–00–00	Brazil	BR_1960–2006
MG747592.1	Long	2004–00–00	Brazil	BR_1960–2006
MG747593.1	Small	2006–00–00	Brazil	BR_1960–2006
MG747594.1	Medium	2006–00–00	Brazil	BR_1960–2006
MG747595.1	Long	2006–00–00	Brazil	BR_1960–2006
MG747596.1	Small	2006–00–00	Brazil	BR_1960–2006
MG747597.1	Medium	2006–00–00	Brazil	BR_1960–2006
MG747598.1	Long	2006–00–00	Brazil	BR_1960–2006
MG747599.1	Small	2006–00–00	Brazil	BR_1960–2006
MG747600.1	Medium	2006–00–00	Brazil	BR_1960–2006
MG747601.1	Long	2006–00–00	Brazil	BR_1960–2006
KF697145.1	Medium	2007–00–00	Peru_MadreDios	MDDV prototype
KF697146.1	Small	2007–00–00	Peru_MadreDios	MDDV prototype
KF697147.1	Long	2007–00–00	Peru_MadreDios	MDDV prototype
KP795084.1	Long	2008–03–00	Peru	Peru-Ecuador_2008–2016
KP795085.1	Medium	2008–03–00	Peru	Peru-Ecuador_2008–2016
KP795086.1	Small	2008–03–00	Peru	Peru-Ecuador_2008–2016
KP691618.1	Long	2009–06–00	Brazil	BR_2009–2018
KP691619.1	Medium	2009–06–00	Brazil	BR_2009–2018
KP691620.1	Small	2009–06–00	Brazil	BR_2009–2018
KP691621.1	Long	2009–06–00	Brazil	BR_2009–2018
KP691622.1	Medium	2009–06–00	Brazil	BR_2009–2018
KP691623.1	Small	2009–06–00	Brazil	BR_2009–2018
OP407852.1	Long	2009–06–00	Brazil	BR_2009–2018
OP407853.1	Medium	2009–06–00	Brazil	BR_2009–2018
OP407854.1	Small	2009–06–00	Brazil	BR_2009–2018

Accession number ID	Genome segment	Collection date	Location	Major groups classification
KP691603.1	Long	2009–07–00	Brazil	BR_2009–2018
KP691604.1	Medium	2009–07–00	Brazil	BR_2009–2018
KP691605.1	Small	2009–07–00	Brazil	BR_2009–2018
KP691606.1	Long	2009–07–00	Brazil	BR_2009–2018
KP691607.1	Medium	2009–07–00	Brazil	BR_2009–2018
KP691608.1	Small	2009–07–00	Brazil	BR_2009–2018
KP691609.1	Long	2009–07–00	Brazil	BR_2009–2018
KP691610.1	Medium	2009–07–00	Brazil	BR_2009–2018
KP691611.1	Small	2009–07–00	Brazil	BR_2009–2018
KP691612.1	Long	2009–07–00	Brazil	BR_2009–2018
KP691613.1	Medium	2009–07–00	Brazil	BR_2009–2018
KP691614.1	Small	2009–07–00	Brazil	BR_2009–2018
KP691615.1	Long	2009–07–00	Brazil	BR_2009–2018
KP691616.1	Medium	2009–07–00	Brazil	BR_2009–2018
KP691617.1	Small	2009–07–00	Brazil	BR_2009–2018
KP691630.1	Long	2009–08–00	Brazil	BR_2009–2018
KP691631.1	Medium	2009–08–00	Brazil	BR_2009–2018
KP691632.1	Small	2009–08–00	Brazil	BR_2009–2018
KP691627.1	Long	2012–00–00	Brazil_MinasGerais_Perdoes	PEDV prototype
KP691628.1	Medium	2012–00–00	Brazil_MinasGerais_Perdoes	PEDV prototype
KP691629.1	Small	2012–00–00	Brazil_MinasGerais_Perdoes	PEDV prototype
MN264267.1	Long	2014–05–00	Haiti	Haiti_2014
MN264268.1	Medium	2014–05–00	Haiti	Haiti_2014
MN264269.1	Small	2014–05–00	Haiti	Haiti_2014
PP154170.1	Small	2015–04–00	Brazil_Amazonas_Tefe	BR_2015–2023
PP154171.1	Medium	2015–04–00	Brazil_Amazonas_Tefe	BR_2015–2023
PP154172.1	Long	2015–04–00	Brazil_Amazonas_Tefe	BR_2015–2023
MF926352.1	Small	2016–04–00	Ecuador	Peru-Ecuador_2008–2016
MF926353.1	Medium	2016–04–00	Ecuador	Peru-Ecuador_2008–2016
MF926354.1	Long	2016–04–00	Ecuador	Peru-Ecuador_2008–2016
MK506818.1	Small	2016–04–00	Ecuador	Peru-Ecuador_2008–2016
MK506819.1	Small	2016–04–00	Ecuador	Peru-Ecuador_2008–2016
MK506820.1	Small	2016–04–00	Ecuador	Peru-Ecuador_2008–2016
MK506821.1	Small	2016–04–00	Ecuador	Peru-Ecuador_2008–2016
MK506822.1	Small	2016–04–00	Ecuador	Peru-Ecuador_2008–2016
MK506823.1	Medium	2016–04–00	Ecuador	Peru-Ecuador_2008–2016
MK506824.1	Medium	2016–04–00	Ecuador	Peru-Ecuador_2008–2016
MK506825.1	Medium	2016–04–00	Ecuador	Peru-Ecuador_2008–2016
MK506826.1	Medium	2016–04–00	Ecuador	Peru-Ecuador_2008–2016
MK506827.1	Medium	2016–04–00	Ecuador	Peru-Ecuador_2008–2016
MK506828.1	Long	2016–04–00	Ecuador	Peru-Ecuador_2008–2016
MK506829.1	Long	2016–04–00	Ecuador	Peru-Ecuador_2008–2016
MK506830.1	Long	2016–04–00	Ecuador	Peru-Ecuador_2008–2016
MK506831.1	Long	2016–04–00	Ecuador	Peru-Ecuador_2008–2016
MK506832.1	Long	2016–04–00	Ecuador	Peru-Ecuador_2008–2016
MT879228.1	Long	2018–03–00	Brazil	BR_2009–2018
MT879229.1	Medium	2018–03–00	Brazil	BR_2009–2018
MT879230.1	Small	2018–03–00	Brazil	BR_2009–2018
PP153981.1	Small	2022–08–00	Brazil_Roraima_AltoAlegre	BR_2015–2023
PP153982.1	Medium	2022–08–00	Brazil_Roraima_AltoAlegre	BR_2015–2023

Accession number ID	Genome segment	Collection date	Location	Major groups classification
PP153983.1	Long	2022–08–00	Brazil_Roraima_AltoAlegre	BR_2015–2023
PP153977.1	Small	2022–10–00	Brazil_Rondonia_PortoVelho	BR_2015–2023
PP153978.1	Medium	2022–10–00	Brazil_Rondonia_PortoVelho	BR_2015–2023
PP153979.1	Long	2022–10–00	Brazil_Rondonia_PortoVelho	BR_2015–2023
PP154128.1	Small	2022–12–00	Brazil_Amazonas_Manaus	BR_2015–2023
PP154129.1	Medium	2022–12–00	Brazil_Amazonas_Manaus	BR_2015–2023
PP154130.1	Long	2022–12–00	Brazil_Amazonas_Manaus	BR_2015–2023
PP153951.1	Small	2023–01–00	Brazil_Rondonia_PortoVelho	BR_2015–2023
PP153952.1	Medium	2023–01–00	Brazil_Rondonia_PortoVelho	BR_2015–2023
PP153953.1	Long	2023–01–00	Brazil_Rondonia_PortoVelho	BR_2015–2023
PP153975.1	Small	2023–01–00	Brazil_Rondonia_PortoVelho	BR_2015–2023
PP153976.1	Long	2023–01–00	Brazil_Rondonia_PortoVelho	BR_2015–2023
PP153980.1	Medium	2023–01–00	Brazil_Rondonia_PortoVelho	BR_2015–2023
PP154011.1	Small	2023–01–00	Brazil_Roraima_SaoJoaoBaliza	BR_2015–2023
PP154012.1	Medium	2023–01–00	Brazil_Roraima_SaoJoaoBaliza	BR_2015–2023
PP154013.1	Long	2023–01–00	Brazil_Roraima_SaoJoaoBaliza	BR_2015–2023
PP154146.1	Small	2023–01–00	Brazil_Amazonas_Labrea	BR_2015–2023
PP154147.1	Medium	2023–01–00	Brazil_Amazonas_Labrea	BR_2015–2023
PP154148.1	Long	2023–01–00	Brazil_Amazonas_Labrea	BR_2015–2023
PP154149.1	Small	2023–01–00	Brazil_Amazonas_Manicore	BR_2015–2023
PP154150.1	Medium	2023–01–00	Brazil_Amazonas_Manicore	BR_2015–2023
PP154151.1	Long	2023–01–00	Brazil_Amazonas_Manicore	BR_2015–2023
PP153966.1	Small	2023–02–00	Brazil_Rondonia_PortoVelho	BR_2015–2023
PP153967.1	Medium	2023–02–00	Brazil_Rondonia_PortoVelho	BR_2015–2023
PP153968.1	Long	2023–02–00	Brazil_Rondonia_PortoVelho	BR_2015–2023
PP153969.1	Small	2023–02–00	Brazil_Rondonia_PortoVelho	BR_2015–2023
PP153970.1	Medium	2023–02–00	Brazil_Rondonia_PortoVelho	BR_2015–2023
PP153971.1	Long	2023–02–00	Brazil_Rondonia_PortoVelho	BR_2015–2023
PP153972.1	Small	2023–02–00	Brazil_Rondonia_PortoVelho	BR_2015–2023
PP153973.1	Medium	2023–02–00	Brazil_Rondonia_PortoVelho	BR_2015–2023
PP153974.1	Long	2023–02–00	Brazil_Rondonia_PortoVelho	BR_2015–2023
PP153993.1	Small	2023–02–00	Brazil_Roraima_Rorainopolis	BR_2015–2023
PP153994.1	Medium	2023–02–00	Brazil_Roraima_Rorainopolis	BR_2015–2023
PP153995.1	Long	2023–02–00	Brazil_Roraima_Rorainopolis	BR_2015–2023
PP153996.1	Medium	2023–02–00	Brazil_Roraima_SaoJoaoBaliza	BR_2015–2023
PP153997.1	Small	2023–02–00	Brazil_Roraima_SaoJoaoBaliza	BR_2015–2023
PP153998.1	Long	2023–02–00	Brazil_Roraima_SaoJoaoBaliza	BR_2015–2023
PP153999.1	Small	2023–02–00	Brazil_Roraima_Rorainopolis	BR_2015–2023
PP154000.1	Medium	2023–02–00	Brazil_Roraima_Rorainopolis	BR_2015–2023
PP154001.1	Long	2023–02–00	Brazil_Roraima_Rorainopolis	BR_2015–2023
PP154002.1	Small	2023–02–00	Brazil_Roraima_Rorainopolis	BR_2015–2023
PP154003.1	Medium	2023–02–00	Brazil_Roraima_Rorainopolis	BR_2015–2023
PP154004.1	Long	2023–02–00	Brazil_Roraima_Rorainopolis	BR_2015–2023
PP154005.1	Small	2023–02–00	Brazil_Roraima_Rorainopolis	BR_2015–2023
PP154006.1	Medium	2023–02–00	Brazil_Roraima_Rorainopolis	BR_2015–2023
PP154007.1	Long	2023–02–00	Brazil_Roraima_Rorainopolis	BR_2015–2023
PP154008.1	Small	2023–02–00	Brazil_Roraima_Rorainopolis	BR_2015–2023
PP154009.1	Medium	2023–02–00	Brazil_Roraima_Rorainopolis	BR_2015–2023
PP154010.1	Long	2023–02–00	Brazil_Roraima_Rorainopolis	BR_2015–2023
PP154014.1	Small	2023–02–00	Brazil_Roraima_Canta	BR_2015–2023
PP154015.1	Medium	2023–02–00	Brazil_Roraima_Canta	BR_2015–2023
PP154016.1	Long	2023–02–00	Brazil_Roraima_Canta	BR_2015–2023
PP154131.1	Small	2023–02–00	Brazil_Amazonas_Labrea	BR_2015–2023
PP154132.1	Medium	2023–02–00	Brazil_Amazonas_Labrea	BR_2015–2023
PP154133.1	Long	2023–02–00	Brazil_Amazonas_Labrea	BR_2015–2023
PP154134.1	Small	2023–02–00	Brazil_Amazonas_Labrea	BR_2015–2023
PP154135.1	Medium	2023–02–00	Brazil_Amazonas_Labrea	BR_2015–2023
PP154136.1	Long	2023–02–00	Brazil_Amazonas_Labrea	BR_2015–2023
PP154137.1	Small	2023–02–00	Brazil_Amazonas_Labrea	BR_2015–2023
PP154138.1	Medium	2023–02–00	Brazil_Amazonas_Labrea	BR_2015–2023
PP154139.1	Long	2023–02–00	Brazil_Amazonas_Labrea	BR_2015–2023
PP154140.1	Small	2023–02–00	Brazil_Amazonas_Manicore	BR_2015–2023
PP154141.1	Medium	2023–02–00	Brazil_Amazonas_Manicore	BR_2015–2023
PP154142.1	Long	2023–02–00	Brazil_Amazonas_Manicore	BR_2015–2023
PP154143.1	Small	2023–02–00	Brazil_Amazonas_Manicore	BR_2015–2023
PP154144.1	Medium	2023–02–00	Brazil_Amazonas_Manicore	BR_2015–2023
PP154145.1	Long	2023–02–00	Brazil_Amazonas_Manicore	BR_2015–2023
PP153948.1	Small	2023–03–00	Brazil_Rondonia_PortoVelho	BR_2015–2023

Accession number ID	Genome segment	Collection date	Location	Major groups classification
PP153949.1	Medium	2023–03–00	Brazil_Rondonia_PortoVelho	BR_2015–2023
PP153950.1	Long	2023–03–00	Brazil_Rondonia_PortoVelho	BR_2015–2023
PP153954.1	Small	2023–03–00	Brazil_Rondonia_PortoVelho	BR_2015–2023
PP153955.1	Medium	2023–03–00	Brazil_Rondonia_PortoVelho	BR_2015–2023
PP153956.1	Long	2023–03–00	Brazil_Rondonia_PortoVelho	BR_2015–2023
PP153957.1	Small	2023–03–00	Brazil_Rondonia_PortoVelho	BR_2015–2023
PP153958.1	Medium	2023–03–00	Brazil_Rondonia_PortoVelho	BR_2015–2023
PP153959.1	Long	2023–03–00	Brazil_Rondonia_PortoVelho	BR_2015–2023
PP153960.1	Small	2023–03–00	Brazil_Rondonia_PortoVelho	BR_2015–2023
PP153961.1	Medium	2023–03–00	Brazil_Rondonia_PortoVelho	BR_2015–2023
PP153962.1	Long	2023–03–00	Brazil_Rondonia_PortoVelho	BR_2015–2023
PP153963.1	Small	2023–03–00	Brazil_Rondonia_PortoVelho	BR_2015–2023
PP153964.1	Medium	2023–03–00	Brazil_Rondonia_PortoVelho	BR_2015–2023
PP153965.1	Long	2023–03–00	Brazil_Rondonia_PortoVelho	BR_2015–2023
PP154017.1	Small	2023–03–00	Brazil_Rondonia_ColoradodoOeste	BR_2015–2023
PP154018.1	Medium	2023–03–00	Brazil_Rondonia_ColoradodoOeste	BR_2015–2023
PP154019.1	Long	2023–03–00	Brazil_Rondonia_ColoradodoOeste	BR_2015–2023
PP154023.1	Small	2023–03–00	Brazil_Rondonia_MonteNegro	BR_2015–2023
PP154024.1	Medium	2023–03–00	Brazil_Rondonia_MonteNegro	BR_2015–2023
PP154025.1	Long	2023–03–00	Brazil_Rondonia_MonteNegro	BR_2015–2023
PP154026.1	Small	2023–03–00	Brazil_Rondonia_MonteNegro	BR_2015–2023
PP154027.1	Medium	2023–03–00	Brazil_Rondonia_MonteNegro	BR_2015–2023
PP154028.1	Long	2023–03–00	Brazil_Rondonia_MonteNegro	BR_2015–2023
PP154029.1	Small	2023–03–00	Brazil_Rondonia_Corumbiara	BR_2015–2023
PP154030.1	Medium	2023–03–00	Brazil_Rondonia_Corumbiara	BR_2015–2023
PP154031.1	Long	2023–03–00	Brazil_Rondonia_Corumbiara	BR_2015–2023
PP154032.1	Small	2023–03–00	Brazil_Rondonia_Corumbiara	BR_2015–2023
PP154033.1	Medium	2023–03–00	Brazil_Rondonia_Corumbiara	BR_2015–2023
PP154034.1	Long	2023–03–00	Brazil_Rondonia_Corumbiara	BR_2015–2023
PP154152.1	Small	2023–03–00	Brazil_Acre_RioBranco	BR_2015–2023
PP154153.1	Medium	2023–03–00	Brazil_Acre_RioBranco	BR_2015–2023
PP154154.1	Long	2023–03–00	Brazil_Acre_RioBranco	BR_2015–2023
PP154155.1	Small	2023–03–00	Brazil_Acre_RioBranco	BR_2015–2023
PP154156.1	Medium	2023–03–00	Brazil_Acre_RioBranco	BR_2015–2023
PP154157.1	Long	2023–03–00	Brazil_Acre_RioBranco	BR_2015–2023
PP154158.1	Small	2023–03–00	Brazil_Acre_PortoAcre	BR_2015–2023
PP154159.1	Medium	2023–03–00	Brazil_Acre_PortoAcre	BR_2015–2023
PP154160.1	Long	2023–03–00	Brazil_Acre_PortoAcre	BR_2015–2023
PP154161.1	Small	2023–03–00	Brazil_Acre_Acrelandia	BR_2015–2023
PP154162.1	Medium	2023–03–00	Brazil_Acre_Acrelandia	BR_2015–2023
PP154163.1	Long	2023–03–00	Brazil_Acre_Acrelandia	BR_2015–2023
PP154164.1	Small	2023–03–00	Brazil_Acre_Acrelandia	BR_2015–2023
PP154165.1	Medium	2023–03–00	Brazil_Acre_Acrelandia	BR_2015–2023
PP154166.1	Long	2023–03–00	Brazil_Acre_Acrelandia	BR_2015–2023
PP154167.1	Small	2023–03–00	Brazil_Acre_Acrelandia	BR_2015–2023
PP154168.1	Medium	2023–03–00	Brazil_Acre_Acrelandia	BR_2015–2023
PP154169.1	Long	2023–03–00	Brazil_Acre_Acrelandia	BR_2015–2023
PP154020.1	Small	2023–04–00	Brazil_Rondonia_CostaMarques	BR_2015–2023
PP154021.1	Medium	2023–04–00	Brazil_Rondonia_CostaMarques	BR_2015–2023
PP154022.1	Long	2023–04–00	Brazil_Rondonia_CostaMarques	BR_2015–2023
PP154035.1	Small	2023–04–00	Brazil_Rondonia_SaoFranciscodoGuapore	BR_2015–2023
PP154036.1	Medium	2023–04–00	Brazil_Rondonia_SaoFranciscodoGuapore	BR_2015–2023
PP154037.1	Long	2023–04–00	Brazil_Rondonia_SaoFranciscodoGuapore	BR_2015–2023
PP153945.1	Small	2023–05–00	Brazil_Rondonia_PortoVelho	BR_2015–2023
PP153946.1	Medium	2023–05–00	Brazil_Rondonia_PortoVelho	BR_2015–2023
PP153947.1	Long	2023–05–00	Brazil_Rondonia_PortoVelho	BR_2015–2023
PP154038.1	Small	2023–05–00	Brazil_Rondonia_Cacaulandia	BR_2015–2023
PP154039.1	Medium	2023–05–00	Brazil_Rondonia_Cacaulandia	BR_2015–2023
PP154040.1	Long	2023–05–00	Brazil_Rondonia_Cacaulandia	BR_2015–2023
PP153984.1	Small	2023–08–00	Brazil_Roraima_Mucajai	BR_2015–2023
PP153985.1	Medium	2023–08–00	Brazil_Roraima_Mucajai	BR_2015–2023
PP153986.1	Long	2023–08–00	Brazil_Roraima_Mucajai	BR_2015–2023
PP153987.1	Small	2023–08–00	Brazil_Roraima_Mucajai	BR_2015–2023
PP153988.1	Medium	2023–08–00	Brazil_Roraima_Mucajai	BR_2015–2023
PP153989.1	Long	2023–08–00	Brazil_Roraima_Mucajai	BR_2015–2023
PP153990.1	Small	2023–08–00	Brazil_Roraima_Mucajai	BR_2015–2023
PP153991.1	Medium	2023–08–00	Brazil_Roraima_Mucajai	BR_2015–2023
PP153992.1	Long	2023–08–00	Brazil_Roraima_Mucajai	BR_2015–2023

Accession number ID	Genome segment	Collection date	Location	Major groups classification
PP154119.1	Small	2023–11–00	Brazil_Amazonas_Manaus	BR_2015–2023
PP154120.1	Medium	2023–11–00	Brazil_Amazonas_Manaus	BR_2015–2023
PP154121.1	Long	2023–11–00	Brazil_Amazonas_Manaus	BR_2015–2023
PP154122.1	Small	2023–11–00	Brazil_Amazonas_Manaus	BR_2015–2023
PP154123.1	Medium	2023–11–00	Brazil_Amazonas_Manaus	BR_2015–2023
PP154124.1	Long	2023–11–00	Brazil_Amazonas_Manaus	BR_2015–2023
PP154125.1	Small	2023–11–00	Brazil_Amazonas_Manaus	BR_2015–2023
PP154126.1	Medium	2023–11–00	Brazil_Amazonas_Manaus	BR_2015–2023
PP154127.1	Long	2023–11–00	Brazil_Amazonas_Manaus	BR_2015–2023
PP154041.1	Small	2023–12–00	Brazil_Amazonas_Manaus	BR_2015–2023
PP154042.1	Medium	2023–12–00	Brazil_Amazonas_Manaus	BR_2015–2023
PP154043.1	Long	2023–12–00	Brazil_Amazonas_Manaus	BR_2015–2023
PP154044.1	Small	2023–12–00	Brazil_Amazonas_Manaus	BR_2015–2023
PP154045.1	Medium	2023–12–00	Brazil_Amazonas_Manaus	BR_2015–2023
PP154046.1	Long	2023–12–00	Brazil_Amazonas_Manaus	BR_2015–2023
PP154047.1	Small	2023–12–00	Brazil_Amazonas_Manaus	BR_2015–2023
PP154048.1	Medium	2023–12–00	Brazil_Amazonas_Manaus	BR_2015–2023
PP154049.1	Long	2023–12–00	Brazil_Amazonas_Manaus	BR_2015–2023
PP154050.1	Small	2023–12–00	Brazil_Amazonas_Manaus	BR_2015–2023
PP154051.1	Medium	2023–12–00	Brazil_Amazonas_Manaus	BR_2015–2023
PP154052.1	Long	2023–12–00	Brazil_Amazonas_Manaus	BR_2015–2023
PP154053.1	Small	2023–12–00	Brazil_Amazonas_Manaus	BR_2015–2023
PP154054.1	Medium	2023–12–00	Brazil_Amazonas_Manaus	BR_2015–2023
PP154055.1	Long	2023–12–00	Brazil_Amazonas_Manaus	BR_2015–2023
PP154056.1	Small	2023–12–00	Brazil_Amazonas_Manaus	BR_2015–2023
PP154057.1	Medium	2023–12–00	Brazil_Amazonas_Manaus	BR_2015–2023
PP154058.1	Long	2023–12–00	Brazil_Amazonas_Manaus	BR_2015–2023
PP154059.1	Small	2023–12–00	Brazil_Amazonas_Manaus	BR_2015–2023
PP154060.1	Medium	2023–12–00	Brazil_Amazonas_Manaus	BR_2015–2023
PP154061.1	Long	2023–12–00	Brazil_Amazonas_Manaus	BR_2015–2023
PP154062.1	Small	2023–12–00	Brazil_Amazonas_Manaus	BR_2015–2023
PP154063.1	Medium	2023–12–00	Brazil_Amazonas_Manaus	BR_2015–2023
PP154064.1	Long	2023–12–00	Brazil_Amazonas_Manaus	BR_2015–2023
PP154065.1	Small	2023–12–00	Brazil_Amazonas_Manaus	BR_2015–2023
PP154066.1	Medium	2023–12–00	Brazil_Amazonas_Manaus	BR_2015–2023
PP154067.1	Long	2023–12–00	Brazil_Amazonas_Manaus	BR_2015–2023
PP154068.1	Small	2023–12–00	Brazil_Amazonas_Manaus	BR_2015–2023
PP154069.1	Medium	2023–12–00	Brazil_Amazonas_Manaus	BR_2015–2023
PP154070.1	Long	2023–12–00	Brazil_Amazonas_Manaus	BR_2015–2023
PP154071.1	Small	2023–12–00	Brazil_Amazonas_Manaus	BR_2015–2023
PP154072.1	Medium	2023–12–00	Brazil_Amazonas_Manaus	BR_2015–2023
PP154073.1	Long	2023–12–00	Brazil_Amazonas_Manaus	BR_2015–2023
PP154074.1	Small	2023–12–00	Brazil_Amazonas_Manaus	BR_2015–2023
PP154075.1	Medium	2023–12–00	Brazil_Amazonas_Manaus	BR_2015–2023
PP154076.1	Long	2023–12–00	Brazil_Amazonas_Manaus	BR_2015–2023
PP154077.1	Small	2023–12–00	Brazil_Amazonas_Manaus	BR_2015–2023
PP154078.1	Medium	2023–12–00	Brazil_Amazonas_Manaus	BR_2015–2023
PP154079.1	Long	2023–12–00	Brazil_Amazonas_Manaus	BR_2015–2023
PP154080.1	Small	2023–12–00	Brazil_Amazonas_Manaus	BR_2015–2023
PP154081.1	Medium	2023–12–00	Brazil_Amazonas_Manaus	BR_2015–2023
PP154082.1	Long	2023–12–00	Brazil_Amazonas_Manaus	BR_2015–2023
PP154083.1	Small	2023–12–00	Brazil_Amazonas_Manaus	BR_2015–2023
PP154084.1	Medium	2023–12–00	Brazil_Amazonas_Manaus	BR_2015–2023
PP154085.1	Long	2023–12–00	Brazil_Amazonas_Manaus	BR_2015–2023
PP154086.1	Small	2023–12–00	Brazil_Amazonas_Manaus	BR_2015–2023
PP154087.1	Medium	2023–12–00	Brazil_Amazonas_Manaus	BR_2015–2023
PP154088.1	Long	2023–12–00	Brazil_Amazonas_Manaus	BR_2015–2023
PP154089.1	Small	2023–12–00	Brazil_Amazonas_Manaus	BR_2015–2023
PP154090.1	Medium	2023–12–00	Brazil_Amazonas_Manaus	BR_2015–2023
PP154091.1	Long	2023–12–00	Brazil_Amazonas_Manaus	BR_2015–2023
PP154092.1	Small	2023–12–00	Brazil_Amazonas_Manaus	BR_2015–2023
PP154093.1	Medium	2023–12–00	Brazil_Amazonas_Manaus	BR_2015–2023
PP154094.1	Long	2023–12–00	Brazil_Amazonas_Manaus	BR_2015–2023
PP154095.1	Small	2023–12–00	Brazil_Amazonas_Manaus	BR_2015–2023
PP154096.1	Medium	2023–12–00	Brazil_Amazonas_Manaus	BR_2015–2023
PP154097.1	Long	2023–12–00	Brazil_Amazonas_Manaus	BR_2015–2023
PP154098.1	Small	2023–12–00	Brazil_Amazonas_Manaus	BR_2015–2023
PP154099.1	Medium	2023–12–00	Brazil_Amazonas_Manaus	BR_2015–2023

Accession number ID	Genome segment	Collection date	Location	Major groups classification
PP154100.1	Long	2023–12–00	Brazil_Amazonas_Manaus	BR_2015–2023
PP154101.1	Small	2023–12–00	Brazil_Amazonas_Manaus	BR_2015–2023
PP154102.1	Medium	2023–12–00	Brazil_Amazonas_Manaus	BR_2015–2023
PP154103.1	Long	2023–12–00	Brazil_Amazonas_Manaus	BR_2015–2023
PP154104.1	Small	2023–12–00	Brazil_Amazonas_Manaus	BR_2015–2023
PP154105.1	Medium	2023–12–00	Brazil_Amazonas_Manaus	BR_2015–2023
PP154106.1	Long	2023–12–00	Brazil_Amazonas_Manaus	BR_2015–2023
PP154107.1	Small	2023–12–00	Brazil_Amazonas_Manaus	BR_2015–2023
PP154108.1	Medium	2023–12–00	Brazil_Amazonas_Manaus	BR_2015–2023
PP154109.1	Long	2023–12–00	Brazil_Amazonas_Manaus	BR_2015–2023
PP154110.1	Small	2023–12–00	Brazil_Amazonas_Manaus	BR_2015–2023
PP154111.1	Medium	2023–12–00	Brazil_Amazonas_Manaus	BR_2015–2023
PP154112.1	Long	2023–12–00	Brazil_Amazonas_Manaus	BR_2015–2023
PP154113.1	Small	2023–12–00	Brazil_Amazonas_Manaus	BR_2015–2023
PP154114.1	Medium	2023–12–00	Brazil_Amazonas_Manaus	BR_2015–2023
PP154115.1	Long	2023–12–00	Brazil_Amazonas_Manaus	BR_2015–2023
PP154116.1	Small	2023–12–00	Brazil_Amazonas_Manaus	BR_2015–2023
PP154117.1	Medium	2023–12–00	Brazil_Amazonas_Manaus	BR_2015–2023
PP154118.1	Long	2023–12–00	Brazil_Amazonas_Manaus	BR_2015–2023
LEIAL2076_ES01_M	Medium	2024–04–00	Brazil_EspiritoSanto_Colatina	ES_2024
LEIAL2076_ES01_S	Small	2024–04–00	Brazil_EspiritoSanto_Colatina	ES_2024
LEIAL2077_ES02_M	Medium	2024–04–00	Brazil_EspiritoSanto_Colatina	ES_2024
LEIAL2077_ES02_S	Small	2024–04–00	Brazil_EspiritoSanto_Colatina	ES_2024
LEIAL2078_ES03_M	Medium	2024–04–00	Brazil_EspiritoSanto_RioBananal	ES_2024
LEIAL2078_ES03_S	Small	2024–04–00	Brazil_EspiritoSanto_RioBananal	ES_2024
LEIAL2079_ES04_M	Medium	2024–03–00	Brazil_EspiritoSanto_RioBananal	ES_2024
LEIAL2079_ES04_S	Small	2024–03–00	Brazil_EspiritoSanto_RioBananal	ES_2024
LEIAL2080_ES05_M	Medium	2024–04–00	Brazil_EspiritoSanto_LaranjaTerra	ES_2024
LEIAL2080_ES05_S	Small	2024–04–00	Brazil_EspiritoSanto_LaranjaTerra	ES_2024
LEIAL2081_ES06_M	Medium	2024–04–00	Brazil_EspiritoSanto_LaranjaTerra	ES_2024
LEIAL2081_ES06_S	Small	2024–04–00	Brazil_EspiritoSanto_LaranjaTerra	ES_2024
321559213_L	Long	2024–04–00	Brazil_EspiritoSanto_Colatina	ES_2024
321559213_M	Medium	2024–04–00	Brazil_EspiritoSanto_Colatina	ES_2024
321559213_S	Small	2024–04–00	Brazil_EspiritoSanto_Colatina	ES_2024

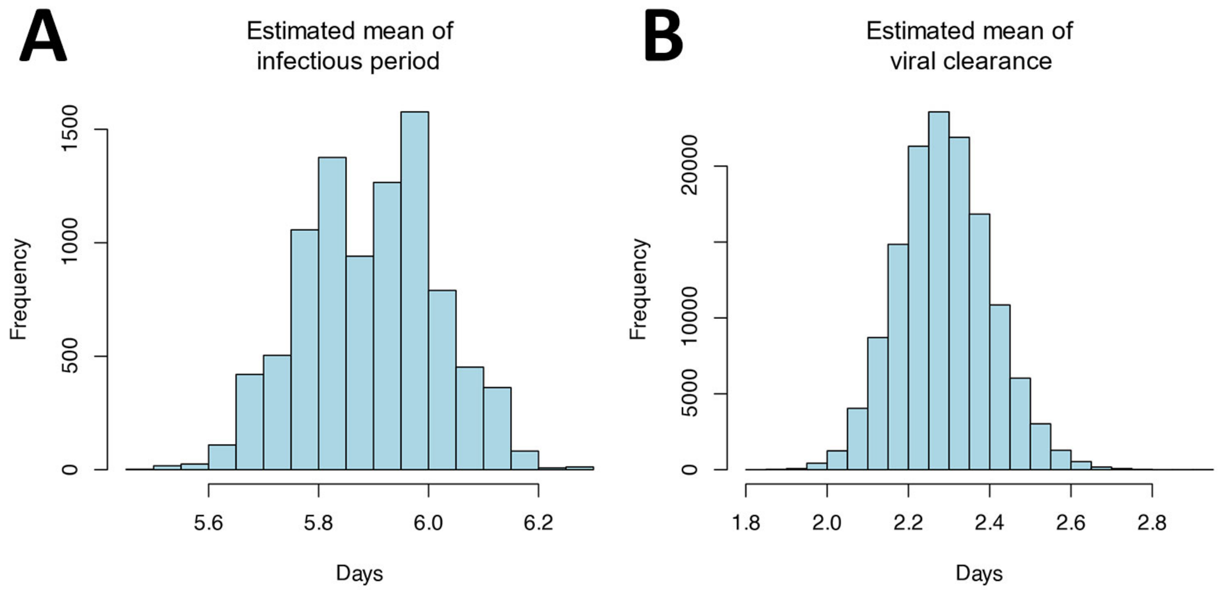

Appendix Figure 1. Histograms of estimated means of key epidemiologic parameters. The left panel represents the estimated mean of the infectious period (in days), while the right panel shows the estimated mean of viral clearance time (in days). Frequencies represent the distribution of parameter estimates obtained from the model-fitting process.

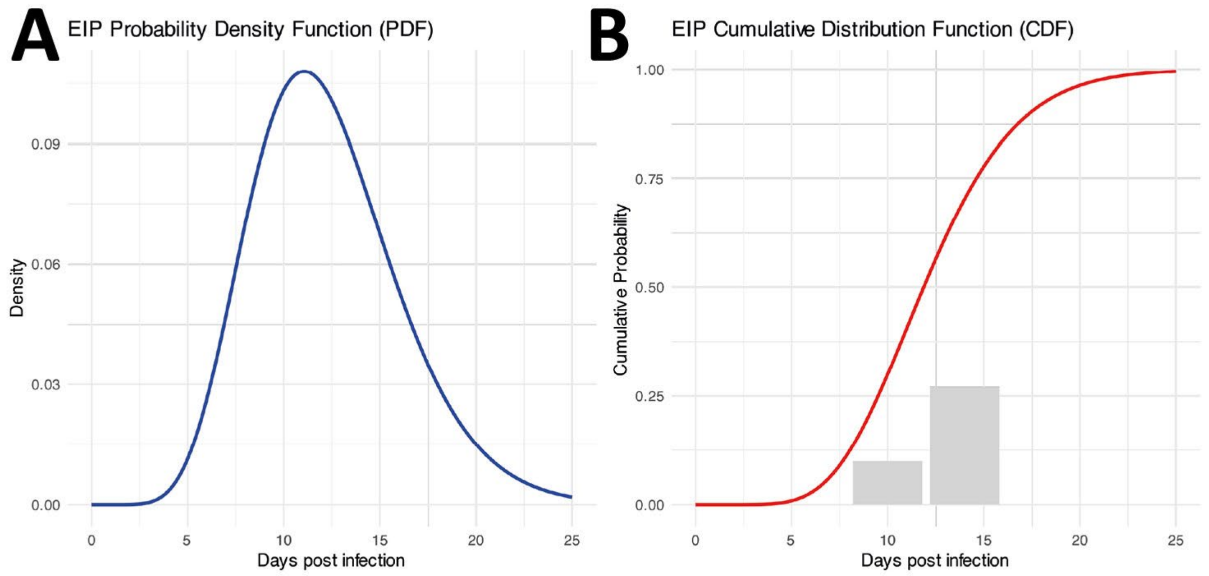

Appendix Figure 2. Maximum likelihood EIP probability density function (left) and cumulative distribution function (right). The aggregated proportion of mosquitoes that transmitted the virus at the relative days post-infection are shown as bars.

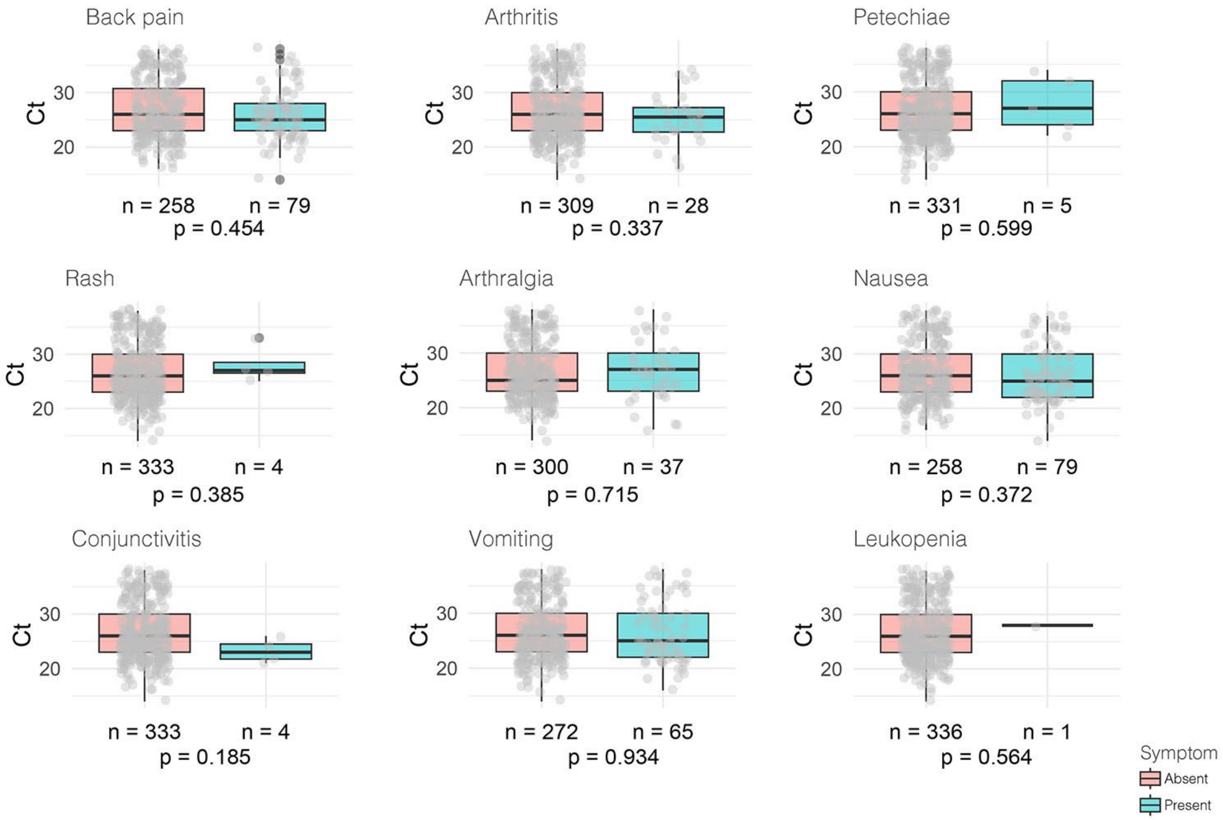

Appendix Figure 3. The boxplots compare the Ct values of individuals with and without each symptom. Horizontal bars represent the Ct medians and interquartile ranges (IQR). Two-sided *p*-values for the nonparametric Mann–Whitney test are shown for each group.
